# A Systematic Review of Real-Time Medical Simulations with Soft-Tissue Deformation: Computational Approaches, Interaction Devices, System Architectures, and Clinical Validations

**DOI:** 10.1155/2020/5039329

**Published:** 2020-02-19

**Authors:** Tan-Nhu Nguyen, Marie-Christine Ho Ba Tho, Tien-Tuan Dao

**Affiliations:** Sorbonne University, Université de Technologie de Compiègne, CNRS, UMR 7338 Biomechanics and Bioengineering, Centre de Recherche Royallieu, CS 60 319 Compiègne, France

## Abstract

Simulating deformations of soft tissues is a complex engineering task, and it is even more difficult when facing the constraint between computation speed and system accuracy. However, literature lacks of a holistic review of all necessary aspects (computational approaches, interaction devices, system architectures, and clinical validations) for developing an effective system of soft-tissue simulations. This paper summarizes and analyses recent achievements of resolving these issues to estimate general trends and weakness for future developments. A systematic review process was conducted using the PRISMA protocol with three reliable scientific search engines (ScienceDirect, PubMed, and IEEE). Fifty-five relevant papers were finally selected and included into the review process, and a quality assessment procedure was also performed on them. The computational approaches were categorized into mesh, meshfree, and hybrid approaches. The interaction devices concerned about combination between virtual surgical instruments and force-feedback devices, 3D scanners, biomechanical sensors, human interface devices, 3D viewers, and 2D/3D optical cameras. System architectures were analysed based on the concepts of system execution schemes and system frameworks. In particular, system execution schemes included distribution-based, multithread-based, and multimodel-based executions. System frameworks are grouped into the input and output interaction frameworks, the graphic interaction frameworks, the modelling frameworks, and the hybrid frameworks. Clinical validation procedures are ordered as three levels: geometrical validation, model behavior validation, and user acceptability/safety validation. The present review paper provides useful information to characterize how real-time medical simulation systems with soft-tissue deformations have been developed. By clearly analysing advantages and drawbacks in each system development aspect, this review can be used as a reference guideline for developing systems of soft-tissue simulations.

## 1. Introduction

In a human body, tissues are commonly classified into hard and soft tissues. While hard tissues do not deform during the motions of human bodies, soft tissues always deform when interacting with themselves, other tissues, and surgical tools. Modeling soft-tissue deformations in an entire organ or only in parts of an organ is still one of the most challenging issues in the biomedical engineering field. In particular, effective integration of soft-tissue deformation behaviors into medical simulation systems has faced two constraints relating to computation speed (or computation time) and system accuracy. Computation speed is the number of computing iterations that a soft-tissue simulation system can be executed in one second on a specific hardware configuration. It is usually measured in frames per second (FPS) or Hertz (Hz). Computation time is a time duration needed to run data acquisition, data pre-/postprocessing, physical behavior simulation, and data visualization in a soft-tissue simulation system. Moreover, two types of accuracies were considered. The first one relates to model accuracy that quantifies the closeness of agreement between the simulated and the real behaviors of soft tissues. The second one deals with the system accuracy that was affected by interaction device accuracy, algorithm accuracy, and model accuracy. Interaction device accuracy is the degree of closeness of the measured values of a physical quantity to its true values. Algorithm accuracy quantifies the correctness of an implemented computational process in relation to the true process. Note that these accuracies should be within the clinically acceptable accuracy bounds for each medical application. In fact, to realistically simulate both geometric deformations and mechanical behaviors of soft tissues within a medical simulation system, computation speed must be in real time [1], and the system accuracy must be within a desired tolerance level according to each medical application. Note that real time is commonly defined as a rate compatible with the graphic animation rate of 30 frames per second (FPS) [2]. Moreover, real time also includes the responding rate of force feedbacks when soft tissues collide with other objects. This rate must be between 100 Hz and 1000 Hz so that human tactile perceptions can feel collisions without interruptions [3]. It is important to note that although real time is one of the most important requirements for clinical applications, most soft-tissue simulation systems hardly satisfied both acceptable model accuracy and real-time computation speed [4]. For instance, Murai et al. stated that the acquisition of internal somatosensory data in real time was crucial because the real time could be used in online diagnosis and assessment processes in surgical applications [5]. Ho et al. showed that the visualization and computing of deformations in real time are essential in surgical simulation of soft tissues [6]. In the field of image-guided surgeries, the estimation of soft-tissue deformations in real time is also one of the most important challenges [7]. Note that in image-guided surgery systems, computation time is commonly expensive due to online data acquisition from medical imaging and additional data processing. In fact, most simulation systems with soft-tissue deformations hardly satisfy real-time requirements [8], and they cannot both correctly compute soft-tissue deformations and effectively achieve real-time computation speeds [7]. However, despite this hard constraint, numerous strategies have been developed for improving both computation speed and accuracy of soft-tissue simulation systems.

Developing soft-tissue simulation systems is a complex engineering task composing of multiple aspects. Each of them has its own contribution to the accuracy and speed of the target system. From system engineering point of view, four important aspects of a real-time medical simulation system include computational approaches, interaction devices, system architectures, and clinical validations. Computational approaches for modeling soft-tissue deformations are first developed according to current requirements about computation speed and system accuracy. It is important to note that the computation speed and system accuracy are mainly affected by the choice of appropriate computational approaches for estimating deformations of soft tissues interacted with external input factors. Interaction devices are then selected to interface between soft-tissue models and real physical environments. This interface needs to be both in real time and in an acceptable accuracy. This requirement often consumes large computation cost from a system. System architectures must also be developed to compromisingly cooperate all system components such as soft-tissue models and input/output interaction devices. On this aspect, system execution schemes and system frameworks should be carefully selected to optimize system performance. Finally, once fully developed, the system must be validated through different validation levels so that it can be used in a target clinical application. Those validation levels include geometrical validation, model validation, system validation, and user acceptability/safety validation. Generally speaking, to simulate soft-tissue deformations in real time while keeping an acceptable realistic level of soft-tissue behaviors, all of the above aspects must be individually and systematically analyzed and developed.

Although the issues of real-time soft tissue simulations were also reviewed, previous review studies rarely analyzed how real-time challenges were solved effectively in a whole system. In particular, all system development aspects should be thoroughly reviewed to describe how both computation speed and system accuracy requirements were achieved. However, the studies just focused on simulating specific types of soft tissues in medical applications, and they did not concern how effectively the real-time constraint was solved. For example, in an interesting review paper proposed by Delingette a full description of realistic soft-tissue modeling in medical simulations was described [9]. However, it just showed out three main problems when realistically simulating soft tissue in medical simulation systems, but the methods for solving those problems were not been analyzed. Other than that, this review was conducted in the year 1998 when technologies were in an initial development stage, so numerous studies that effectively solved the soft-tissue deformation issues have not been analyzed in this review study. Sun et al. [1] also examined a relative diversity of tissue simulation procedures with the help of computer technologies. Although this study covered aspects in the tissue simulation procedure (3D reconstructions, tissue classifications, and clinical applications), it did not focus on soft-tissue modeling and just finished at describing general ideas of each aspect rather than analyzing advantages and disadvantages of methods/algorithms employed in each aspect. Moreover, this study was not answered how the challenge of achieving both real-time computation speeds and acceptable system accuracy was solved. Mainly analyzing advantages and disadvantages of modeling physical deformations, Nealen et al. [10] presented a full description about mathematical functions, explanations of the physical meaning, and analyses of computation results, but they mainly concerned accuracies of each modeling method rather than the computation speed when employed in a specific simulation system. Up to now, with the abundant developments of software/hardware technologies and soft-tissue modeling methods, numerous studies have reasonably proposed effective solutions for both achieving real-time computation speeds and acceptable system accuracy in simulation systems. However, they have not been summarized in a systematic way and analyzed completely to estimate general trends and weakness for future developments. Consequently, to complement those gaps, this review paper is proposed to answer the following questions:

1. How have computational approaches been developed for both achieving real-time computation speed and keeping acceptable system accuracy?
2. Which interaction devices have been interfaced effectively in real-time soft-tissue simulation systems?
3. How have system architectures been developed for cooperating with computational approaches and interaction devices in real time?
4. How have been the real-time soft-tissue simulation systems validated in clinical applications?

Moreover, real-time soft-tissue simulation systems proposed in literature were analysed sequentially and summarized according to four system development aspects: computational approaches, interaction devices, system architectures, and clinical validations. Moreover, trends and gaps of each development aspect were also presented. Recommendations for future researches were finally proposed.

## 2. Materials and Methods

**Figure 1:**
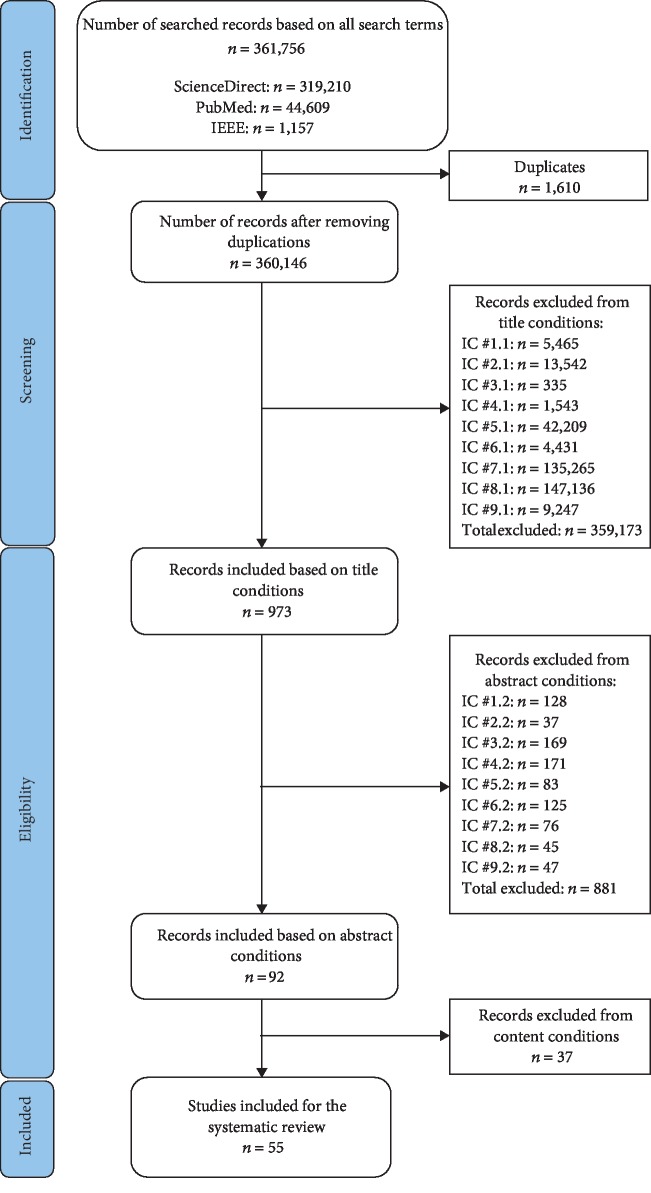
Workflow of the selection process using PRISMA protocol for the performed systematic review.

A systematic review method was conducted using the PRISMA protocol [11] (Figure 1). Three scientific databases were chosen: ScienceDirect, PubMed, and IEEE. In more details, a focus on human soft tissues like upper/lower limb muscles, facial muscles, livers, and skins was done. A special attention was also given on the contributions related to the improvement of computational methods and/or employing effective hardware/software system architectures for real-time medical simulation systems. Finally, other articles focused on analyzing applications of real-time soft-tissue models for system validation, user acceptability and safety requirements were included. Note that in this present review, the method refers to the development strategy of mathematical constitutive formulations of soft-tissue deformations based on a specific computational approach. Reviewed studies relate to mesh-based and meshfree methods. An algorithm concerns the procedure to compute soft-tissue deformations using specific modeling methods. A model refers to the mathematical representation of soft-tissue deformations using mesh-based and meshfree-based methods. A set of search terminologies were defined for the literature investigation, and then, each terminology was presented in a search term by using AND/OR operators. The used search terminologies and their appropriate search terms are listed in Table 1. For the systematic information retrieval process, journal articles published up to December 2017 were assessed.

### 2.1. Selection Methodology

Selection was the most significant procedure for choosing both qualitatively and quantitatively appropriate articles for the systematic review. After identification from the search engines, retrieved articles were automatically saved to their suitable folders using the Mendeley paper management system. Two independent reviewers (TNN and TTD) screened and selected relevant papers for this review study. They also participated into the quality assessment. Consensus discussion was done when necessary for solving disagreements. The number of included/excluded articles is summarized in Table 2. Firstly, the duplicates were checked with the duplication tool in the Mendeley software. The number of duplicated papers at this stage was 1,610 for all search terms. Then, the general and specific eligibility criteria were applied to all unduplicated articles. The title inclusion criteria were first used for filtering out the irrelevant articles. The included articles at this phase were 973, which were then enrolled to the abstract filtering criteria for selecting the most pertinent articles. After reading all the abstracts, 92 included articles were then read in full-text to select the best qualitative and quantitative articles for systematic review. Finally, the number of included articles was 55. Specifically, the flow chart of the selection procedure illustrating the number of included/excluded articles after each selection stage is shown in Figure 1. To answer the identified research questions, the selected 55 papers were categorized into four classes. The first category concerns the computational approaches for modeling deformations of human soft tissues in real time. The second category relates to the disadvantages and advantages of interaction devices for getting the external data from soft tissues and visualizing the processed data. The third category deals with the characteristics of medical hardware/software systems consisting of graphic user interfaces (GUIs), programming languages, programming frameworks, and other techniques for developing soft-tissue simulation systems. The final category composes of system validations in clinical contexts and the analyses of user acceptability and safety requirements of developed systems. Additionally, each selected paper could also be grouped on multiple categories if their contents related to more than one category.

### 2.2. Eligibility Criteria

The inclusion/exclusion criteria were clearly defined based on the meaning of each search terminology. The list of inclusion criteria for each search terminology is shown in Table 3. In addition, to keep the literature at a high academic level, only journal articles were considered for the present review. Moreover, the articles in conferences with a couple of pages are initially eliminated. Other kinds of low-quality written forms such as letters, judgements, and book chapters were also not selected. Other than that, the articles that were not written in English were excluded from the literature review.

### 2.3. Quality Assessment

The quality assessment procedure was established to rate the quality of each analyzed paper. Eighteen yes-no assessment items were defined and used. Papers related to computational approaches bias were evaluated using the following four items: (1) Was the method adequately used/developed and described for the involved tissue behavior? (2) Was the verification well-performed for the used/developed method? (3) Was the validation systematically performed for the used/developed method? (4) Did the method really satisfy the real-time constraint? Papers related to interaction devices bias were evaluated using the following four items: (5) Was the devices well selected for the system? (6) Was the device accuracy adequate for the real-time constraints? (7) Was the device easy enough to use for a clinical routine practice? (8) Is the device price suitable for a clinical setting? Papers related to system architecture bias were evaluated using the following four items: (9) Was the system adequately described? (10) Was the system developed with the participation of the end users? (11) Was the system scalable? (12) Were the system frameworks adequately selected for implementing the system of interest? Papers related to clinical validation bias were evaluated using the following six items: (13) Was the study adequately validated with in vitro data? (14) Was the study adequately validated with in vivo data? (15) Was the study adequately validated with patient data? (16) Was the level of validation suitable for translating the outcomes into clinical routine practices? (17) Was the user acceptability performed for patients? (18) Was the user acceptability performed for clinical experts?

Note that the user acceptability validation is commonly conducted after developing a full-simulation system. This validation targets at validating the acceptability level related to graphic system's user interfaces, system's ease-of-use, system's functions, system's robustness, etc., during short-term and/or long-term evaluation campaigns for clinicians. Regarding the verification of the developed method, an error check list related to the input data, algorithm execution, and output visualization is defined. The “well-performed” category is assigned to a paper if all these three elements are satisfied.

## 3. Results

### 3.1. Overall Quality Assessment Analysis

Statistical results of the quality assessment procedure are presented in Table 4. Overall, most selected articles well described, verified, and validated the computational approaches. Tissue behaviors were well described in selected studies. Over 80% of articles modelled the tissue physical characteristics in the methods while the others just focused on soft-tissue deformations. Most authors all well conducted verifications (76%) and validations steps (89%). For examples, in the study of Cotin et al. [12], after the developed methods are clearly described, the authors designed an example system using the method and analyzed the computed results. Their outputs were compared with other methods and showed a faster computation time and higher accuracy level. Visualizations were also clearly presented to show computed deformations and collisions with a virtual surgical tool. System performance and accuracy were also measured and verified. Thus, the verification was well-performed in this study. The verification procedure was not well-performed in Allard et al. [13] because they mainly introduced the SOFA framework, and the authors just verified their results by visual assessments. Although the real-time constraint was strongly required in the study objectives, only 65% of the developed computational approaches really satisfied this constraint. The others just nearly reached the real-time conditions. For example, the computation frame rates were nearly 30 FPS. In addition, all interaction devices were all accurate enough for use in clinical routines with acceptable prices, and they were also well selected for appropriate computational approaches and system architectures. Moreover, the data transmission bandwidths of these selected devices were relatively much faster than the computational and graphical rendering speeds, so they were all suitable for real-time applications. Over 50% of articles have implemented their developed computational approaches into a simulation system. They also well described the architectures and frameworks of the implemented systems for future developments. However, these systems were rarely developed with the participation of end users. They were mainly tested with the developers and did not have many feedbacks from users. Most of implemented simulation systems could not be directly transferred into the clinical routine practices due to lack of validations with *in vitro*, *in vivo*, and real patient data. The computed results of simulation systems were often validated with *in vitro* data acquired from phantom tissues with physical testing machines. Due to difficulties of acquiring data from living organs, only 13% of studies conducted clinical validations using *in vivo* data. Moreover, only external data such as deformations were available. Finally, the user and expert acceptability aspects were occasionally (i.e., only 4% and 7% of studies) investigated. Note that most developed systems were initially designed for testing and verifying the computational approaches rather than for developing real clinical applications.

### 3.2. Computational Approaches

To achieve real-time computation speed when rendering and computing soft-tissue deformations, two modeling approaches have been commonly adopted. The first approach that we called model development (MD) mainly focuses on geometry discretization strategy and mathematical constitutive formulations of soft-tissue stress-strain relationships. Soft-tissue models developed using this approach are commonly executed with a single-thread platform in a faster and/or more accurate manner. The second approach that we named as constitutive model implementations (MI) relate to the algorithmic implementations of the existing constitutive models using developed methods for soft-tissue deformations onto a more powerful hardware configuration such as Graphic Processing Unit (GPU) system. Thus, systems can compute soft-tissue models faster and more robustly than the traditional ones. Particularly, this concept refers to a family of more suitable programming algorithms to parallelize the execution tasks of a developed modeling method, which was traditionally running on a single-thread platform. For example, Berkley et al. addressed a MD study related to the development of a Linearized FEM (L-FEM) method built from the reduced object kinematics [14]. The L-FEM method is suitable for modeling linear elasticity of soft tissues. This method is faster than the FEM. Moreover, in the study of Joldes et al., the total Lagrangian (TL) formulation was applied to improve the computation speed of the traditional FEM [15]. Additionally, the total Lagrangian explicit dynamic FEM (TLED-FEM) formulation was developed by Miller et al. and it could run faster than the FEM when executing on the same CPU-based platform [16]. Regarding the model implementation (MI) approach, only the implicit time integration of FEM method has been proven to be the most suitable for parallel implementation. This method was implemented in a GPU platform by Taylor et al. [17].

**Figure 2:**
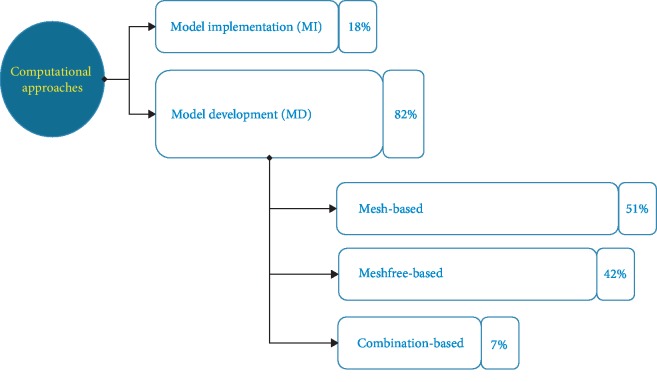
Distribution of computational approaches (MD and MI) and associated techniques for MD approach in the literature.

**Figure 3:**
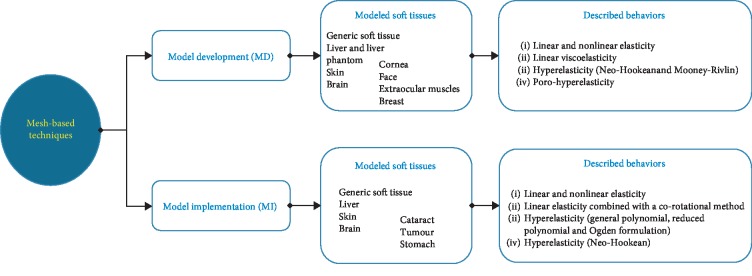
Overview of all modeled soft tissues and different described behaviors for mesh-based studies.

**Figure 4:**
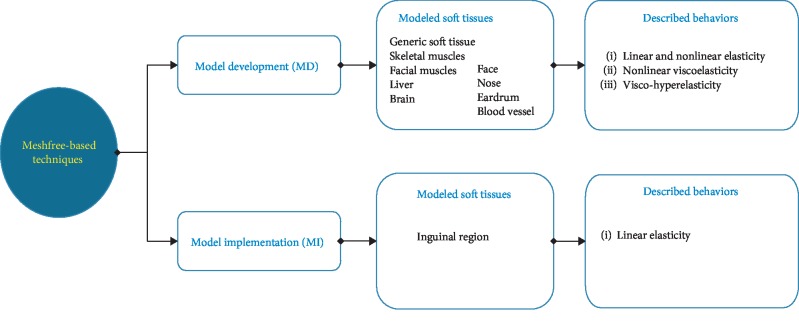
Overview of all modeled soft tissues and different described behaviors for meshfree-based studies.

It is interesting to note that most studies focused at developing new mathematical methods for modeling the soft-tissue deformations rather than implementing the developed modeling methods into a specific hardware configuration to accelerate the computation speed. The distribution of the two approaches throughout the selected literature is illustrated in Figure 2. Obviously, among the total of 55 studies, over 80% of the studies proposed the model development of soft-tissue deformations while only 18% of studies took advantages of specific hardware to accelerate available modeling methods. Regarding the MD approach, we grouped all developed computational methods into three categories: mesh, meshfree, and hybrid modeling methods (Tables 5–7). In more details, the mesh-based modeling methods refer to the development of the finite element method (FEM) and its variations to simulate the soft-tissue deformations in real time (Figure 3). The meshfree-based modeling techniques refer to the decomposition of soft-tissue model into simpler physical submodels or representations without meshing the domains of interest (Figure 4). The hybrid modeling methods take advantage of cooperating multiple modeling methods to increase both computation speeds and model accuracy. The distribution of selected studies according to each modeling method is shown in Figure 2. The result shows that up to 51% of the studies related to the mesh-based methods. The use of the meshfree-based methods reaches over 42%. Finally, the percentage of hybrid methods is around 7%.

### 3.3. Model Development Approaches

#### 3.3.1. Mesh-Based Modelling Methods

**Figure 5:**
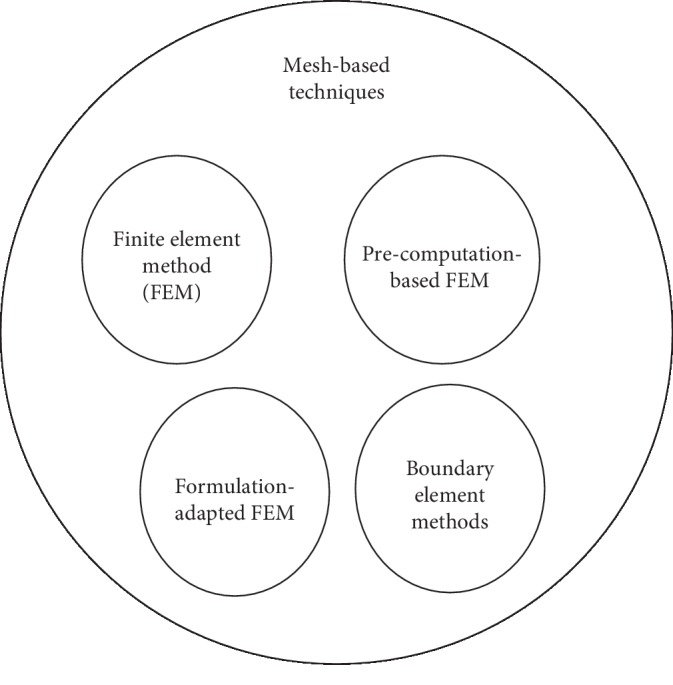
Overview of common computation strategies for mesh-based studies.

Mesh-based modeling methods are grouped into four common computation strategies: the finite element modeling method (FEM), the precomputation-based FEM, the formulation-adapted FEM, and the boundary element methods (Figure 5). Note that in this present review, the term “deformation models” relates to soft-tissue models developed using a specific modeling method while the term “simulation models” refers to numerical models in general meaning.

The finite element method (FEM) has been popularly employed in the literature despite of its very high computational cost. Deformable objects are geometrically meshed by a set of elementary components called finite elements. These elements are connected by nodes whose quantity defines the size of the FE model. Material properties are commonly assigned into each finite element. Then, the physical behavior of solid object deformations is described by a set of constitutive equations. Finally, the resolution of these equations on the nodes with prescribed boundary and loading conditions leads to the stress-strain relationships of the deformable objects. FEM provides a very high level of accuracy and realistic deformations in both linear and nonlinear cases. For example, Wu et al. [30] modeled the facial muscles by FEM to animate the facial expressions. Each single muscle was considered an incompressible and hyperelastic material. Each muscle model includes 1,180 nodes and 28,320 DOF. Note that the computing time could not be achieved in real time. Karami et al. employed also the FEM for modeling the extraocular muscles (EOMs) in an eye to estimate the muscular activations and directions [35]. The eyeball model includes 1,970 nodes and 8,638 elements. Each muscle model includes 1,100 nodes and 2,673 elements. The computation time needed to solve the model was 20 ms.

The precomputation-based FEM is the most popular variation of FEM. This method uses the relationship between the mechanical forces and the deformations precomputed from the accurate FEM with full physical and biomechanical characteristics to train an approximate model. To achieve this goal, a database of the accurate FE simulation outcomes needs to be constructed a priori. The computational accuracy and speed of the simulated model depend on the types of employed approximate techniques such as linear/nonlinear regression functions and machine learning (ML). By using this strategy, Cotin et al. developed a liver surgical simulation system [12]. Sedef et al. provided a solution for real time and realistic FEM for simulating viscoelastic tissue behavior in medical training based on the experimental data collected from a robotic tester [19]. Sela et al. proposed an effective solution for dealing with the topological changes in cutting simulations [20]. Peterlik et al. simulated the human liver with realistic haptic feedback and deformations embedded with both nonlinear geometric and material parameters [3]. Morooka et al. designed a navigation system for the minimally invasive surgeries using a neural network model [31]. Martínez-Martínez et al. used the decision tree and two tree-based ensemble methods for simulating the breast compression [36]. Lorente et al. applied decision trees, random forests, and extremely randomized trees models to simulate biomechanical behaviors of a human liver during the breathing action [8]. Tonutti et al. also applied artificial neural networks (ANNs) and support vector regression (SVR) algorithms for learning the precomputed data from the FEM model of a human tumor [7]. Luboz et al. used a set of pressure frames compressed into a small number of modes by proper orthogonal decomposition [37]. This method allows the summarized modes to be described by a linear set of scalar coefficients, and this reduced set of pressure map modes was then inputted to the FE to compute the strain field modes.

The formulation-adapted FEM has been developed by mathematically alternating the FEM formulations with the other modeling methods. One of them is called linearized FEM (L-FEM) in which the kinematic behavior of the simulated object is linearized to the first order of approximations during a specific timing period. Thus, the FEM model built from the reduced object kinematic is also simplified and executed much faster than the original one. Due to the simplification, the L-FEM is only suitable for modeling the soft tissues with linear elastic materials. For instance, Berkley et al. applied the L-FEM to the virtual suturing application [14]. Moreover, Audette et al. divided a FEM model into multiple submeshes [18]. All submeshes were computed independently in parallel threads of a real-time operating system to output the local deformations. Garcia et al. presented another reduction method called matrix system reduction FEM (MSR-FEM) [21]. The method focused rather on computing the regions of interest than the whole model. The order reduction method (ORM) was developed by Niroomandi et al. to reduce the complex computation of nonlinear FEM for real-time simulations [29]. The total Lagrangian (TL) formulation was also applied in a FE model to improve the computation speed. Joldes et al. used this approach to develop a FE model for an efficient hourglass control application [15]. A variation of this method, called total Lagrangian explicit dynamic FEM (TLED-FEM), was also developed by Miller et al. for an image-guided surgery applications [16]. This method was also employed by Joldes et al. to simulate the deformations of a human brain [15]. They all successfully improved both the sizes and computation speeds of the developed models. Another version of TL-FEM proposed by Marchesseau et al. was called multiplicative Jacobian energy decomposition (MJED) FEM [26]. This approach optimizes the generation of stiffness matrix in TL-FEM to solve the linear system of equations during each iteration. Turkiyyah et al. aimed at physically simulating the mesh cutting in real time thanks to the controlled discontinuities in the basic functions and the fast incremental methods for updating the global deformations [28]. Finally, element-by-element precondition conjugate gradient FEM (EbE PCG-FEM) was developed by Mafi and Sirouspour [32]. This method combined the FEM with a conjugate gradient method by alternating the mesh topological computation at run time by iterations. Thus, the developed model would be faster than the original one using FEM and required less system memories during execution. A new preconditioning technique (pre-cond FEM) was also proposed by Courtecuisse et al. for improving the computational time of soft-tissue deformations [33]. This technique could simulate topologically changes and haptic feedbacks of homogeneous and heterogeneous materials in acceptable accuracy.

The boundary element methods are based on surface deformations to deduce the internal deformations in real time. Monserrat et al. developed a surgery simulation system using this method [4]. Compared with the FEM, the BEM only required the discretization of the object's surface so that it could provide an optimized, fast, and easy implementation. Another surface-based method for developing the soft-tissue models was called Laplacian surface deformation (LSD) was first proposed in Sorkine et al. [41]. The method represented the object surface based on the Laplacian of the mesh. Wang et al. also employed the LSD method for nose surgery in a complete surgical system for automatic individual prosthesis design [48]. Goto et al. used the statistical analysis method (SAM) for detecting features on the facial surface through 2D images, and then, the detected features were mapped to a generic 3D facial model for generating the expressions using the surface deformation method [39]. Moreover, the computation speed of the facial expression estimators was enhanced by using a scaling polygon mesh method based on iterative edge contractions by Bonamico et al. [40]. Chandrasiri et al. proposed a strategy for converting the acquired facial expressions to the MPEG-4 FAP [60], stream to deform the 3D surface facial models robustly and in real time [42]. Wan et al. [49] and Woodward et al. [55] used the landmark-based and muscle-based facial expression estimation to animate the 3D surface facial model. The used methods were radial basic function (RBF) and geodesic distance. Le et al. took advantages of the thin-shell linear deformation model to reconstruct the facial pose via the facial marker displacements [50].

#### 3.3.2. Meshfree-Based Modelling Methods

Compared to the mesh-based modeling methods, meshfree-based modeling methods use discrete points for representing continuum, and it takes advantages of interpolation methods to solve the partial differential equations (PDEs) [59]. Thus, a simulated soft-tissue object is commonly modeled as a distribution of discrete nodes inside to form a complete volumetric model. These nodes are embedded with a shape function to form the model's stiffness matrix and to describe biomechanical characteristics of the soft-tissue object [56]. In particular, this approach does not need to preprocess all cell elements to estimate the global deformations like mesh-based modeling methods do. Consequently, the meshfree-based modeling techniques are much faster than the mesh-based modeling strategies, and they can simulate large deformations in real time. Because of these advantages, the meshfree-based modeling methods have received much attentions from research community in the recent years. One of the most popular methods using the meshfree-based strategy is the mass-spring system modeling (MSM) method. Nedel et al. applied the MSM method to model the muscle deformations in real time [38]. Brown et al. applied the MSM method for a surgical training system [2]. Chen et al. also used the MSM for developing a deformable model for haptic surgery simulation [44]. The MSM was also applied to simulate the 3D model of the human inguinal region by López-Cano et al. [45]. Ho et al. developed a deformable tympanic membrane using the MSM method for simulating the real-time deformation and cutting in a virtual reality myringotomy simulator [6]. Another well-known method of meshfree-based method is called the mass tensor method (MTM) in which the modeled object is approximated into a tetrahedron mesh. Inside each tetrahedron in the MTM, the displacement vectors of four vertices are linearly interpolated into the displacement field of this tetrahedron [57]. The MTM was used by Mollemans et al. to simulate the soft-tissue deformations after bone displacement [43]. An improvement of MSM called mass-spring-damper (MSD) modeling methods was proposed by Basafa and Farahmand [47]. The results illustrated that with a simple cube model including 96 nodes and 270 tetrahedrons, the computation time was just about 0.005 s for each step. Another improvement of MSM was developed by Goulette et al. called hyperelastic mass links (HEML) in which the forces at a specific node are considered a sum of force functions from the neighboring nodes connected with it [53]. Experiments showed that with the 21,436-tetrahedron HEML model, the computation time was at 21.24 ms corresponding with 47 FPS. A different aspect for meshfree-based methods is proposed by Lim and De known as the point collocation-based method of finite sphere (PCMFS) [46]. The technique was based on the combination between the multiresolution approaches and the fast analysis strategies for nonlinear deformations for the active regions where being contacted by the surgical tool tip. A distinctive modeling method for meshfree-based method was inverse dynamic computation (IDC) proposed by Murai et al. for the musculoskeletal system [5]. Zhang et al. developed an elastic-plus-muscle-distribution-based (E+MD) to model the facial muscle distribution for generating the facial expressions in real time [51]. Another method called the time-saving volume-energy conserved ChainMail (TSVE-ChainMail) was proposed by Zhang et al. [54]. The method was developed from the traditional ChainMail method in which the model is represented as a spring system. Zhou et al. have also proposed a Marquardt radial basis meshless method (MRM) for the soft-tissue cutting [56]. In addition to these studies, it is important to note that a large range of soft-tissue models (brain, ligament, and atrioventricular valves) were also developed using the element-free Galerkin method and isogeometric method [61–65]. Due to the used keywords, this present review does not include these works. Thus, interested readers could use more specific keywords to get information about these methods.

#### 3.3.3. Hybrid Modelling Methods

Hybrid methods have been intensively investigated in the literature due to its cooperative functions which take advantages from multiple methods. For instance, although the mass-tensor method (MTM) is fast and suitable for simulation of the soft-tissue deformation in real time, it still lacked the realistic biomechanical characteristics, especially when simulating the nonlinear materials. On the other hand, the FEM has realistic simulation of biomechanical behaviors of soft tissues, but it has high computation cost. Additionally, the precomputation-based methods (pre-comp FEM) have very high performances for simulating the soft-tissue deformations in real time based on the precomputed data from the FEM, but they cannot handle the topological changes. Consequently, the combination between MTM, FEM, and pre-comp FEM can not only simulate the deformation in real time but also handle cutting and tearing realistically with nonlinear materials. This approach was first developed by Cotin et al. [57]. The result of this study showed that the update frequency was able to reach at 40 Hz with an MTM having 760 vertices and 4000 edges. Yarnitzky et al. combined the physically kinematic model with the local FE model to estimate the stresses and deformations inside a plantar foot's soft tissues during gait [58]. Allard et al. introduced a well-known SOFA framework supporting biomedical researchers modularly and flexibly to develop new soft-tissue deformation models [13]. The framework was comprised of multiple modeling methods combined effectively to simulate the soft tissues according to their requirement level of real-time constraints. Zhu and Gu also applied multiple modeling methods to develop a hybrid deformable model for real-time surgical simulation [59]. Different cooperative components exist in the system such as boundary the element method (BEM), the mass-spring method (MSM), and a particle surface interpolation algorithm.

### 3.4. Model Implementation Approaches

The model implementation (MI) approach mainly focusses on algorithmic implementation of soft-tissue models based on developed modeling methods onto a more powerful hardware configuration. This approach can improve the computational performance of the developed soft-tissue deformation models, even faster and more robust than the MD approach. In particular, the MI approach mostly aims at finding more suitable programming algorithms to parallelize the execution functions of the soft-tissue deformation models onto a graphic processing unit (GPU) platform rather than onto a central processing unit (CPU) platform. Basically, GPUs are comprised of highly parallel architectures. Each separate GPU contains numerous processors and memory segmentations, and each processor works independently on its own data distribution. Consequently, although the clock frequencies of GPUs are often smaller than CPUs, the overall computation speed of GPUs are much faster than CPUs, even when CPUs can be composed of multiple processing cores up to now. Furthermore, various programming frameworks supported for model implementations have been improved in an easier and flexible ways. Two classical interfaces have been employed for programming on GPUs have been OpenGL, application programming interfaces (APIs), DirectX, CUDA from NVIDA, and CTM from ATI. These frameworks have been written in high-level C-programming language which bring many benefits for modelers to implement their developed methods executing on GPU effectively [17]. An analysis of GPU implementations for surgical simulations was reviewed by Sørensen and Mosegaard [66]. They concluded that GPUs would become much powerful and cost-effective platforms for implementing the soft-tissue deformation models in real-time medical environments. However, to be able to achieve benefits from this implementation approach, the developed modeling methods must be compatible and be able to reconfigure with parallel computations [66]. The first model implementation strategy was proposed by Taylor et al. [17]. The authors implemented a model using the total Lagrangian explicit dynamic (TLED) FEM onto a NVIDIA GeForce 7900 GT GPU platform, and the results showed that the computation speed of the implemented model was much faster than the CPU-implemented model. A human brain model using the TLED-FEM was also implemented on the NVIDIA Tesla C870 GPU platform by Wittek et al., and the computational performance was also accelerated significantly [24]. For instance, with the brain model of 18,000 nodes and 30,000 elements (approximately 50,000 degrees of freedom), the average time for estimating the brain deformations was less than 4 s when implemented on GPU, and the time implemented on the CPU platform was up to 40 s. A model using the explicit FEM in a real-time skin simulator was also implemented on a GPU platform by Lapper et al. and the simulation results could be accelerated to reach real-time goal [25]. Joldes et al. employed a GPU platform using a programming guide NVIDIA Compute Unified Device Architecture (CUDA) to speed up the TLED-FEM human brain model [23]. Strbac et al. also employed the model using TLED-FEM onto multiple General-Purpose Graphics Processing Units (GPGPUs) to evaluate the efficiency of this implementation with the current commercial solutions [34]. The experimental evaluations showed that when the size of the model increased from 125 to 91,125 elements, the computational time was from 1 s to 1 h 39 min 37 s running on Abaqus commercial software, from 0.149 s to 34.143 s running on the most powerful GPU of GTX980. Courtecuisse et al. proposed an implementation, called linearized FEM (L-FEM), which was the combination between the linear elastic material with a FEM [27]. Mafi et al. deployed a model using element-by-element preconditioned conjugate gradient (EbE PCG) FEM method in the GTX470 GPU platform for speeding up the deformation computation in real time [32]. The implicit time integration of a nonlinear FEM on the GPU platform was also performed by Courtecuisse et al. [33].

### 3.5. Interaction Devices

**Figure 6:**
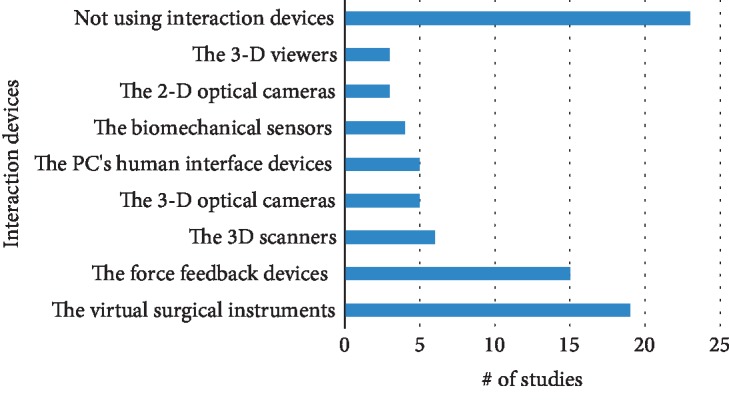
The distribution of using interaction devices in the chosen literature.

In addition to the model development methods and the implementation approaches, the interaction devices contribute significantly to the whole system accuracy and computational time. After user commands are transferred to the computer system through input devices, the computer system must execute the simulated model according to the commanded strategies. Once each simulation iteration is completed, the estimated feedbacks from the simulated model are transmitted to the user through the output devices. Consequently, the total accuracy of both input/output interaction devices and soft-tissue models must be at least equal to the desired accuracy tolerances in each medical application. Different interaction devices have been used in the reviewed studies. However, due to the focused objectives on developing the modeling methods, up to 42% of reviewed studies did not use interaction devices in their simulation systems. The interaction devices in the remaining studies could be divided into different types: the virtual surgical instruments and force-feedback devices, the 3D scanners, the biomechanical sensors, the PC's human interface devices, the 3D viewers, and the 2D and 3D optical cameras. The statistic distribution of used interaction devices is shown in Figure 6. It is clearly showed that the virtual surgical instruments have been popularly used with 19 studies, and the least used device was the 3D viewers and the 2D optical cameras with only 3 studies. The second most popular interaction devices are the force-feedback devices which were found on 15 studies. Other remaining interaction devices have been utilized by only from 4 to 6 studies. In fact, most of the studies have taken advantage of the force-feedback devices always combined with the virtual surgical instruments for interacting with the simulated model. Moreover, other interaction devices certainly used in computer systems, such as computer screens and computer keyboards, are not deeply analyzed in this review paper due to their obvious contributions to the simulation system.

### 3.6. Virtual Surgical Instruments and Force-Feedback Devices

The virtual surgical tools have been widely combined with force-feedback devices to communicate between a user and a simulated model so that the simulation system could become more flexible and realistic. The functions of virtual surgical instruments are to transfer the controlled signals from external real devices to the simulated model and to feedback the calculated biomechanical parameters from the simulated model to the external haptic devices [2, 14, 44, 47]. The speed of transmitting data from/to simulated models must be relatively high so that the visualization and haptic feedback can be simulated realistically [2, 14]. Force-feedback devices are the input/output devices having a function of interfacing between a user and a virtual surgical tool. When the interactions are received from the virtual surgical tool, the simulated model will react and calculate haptic forces during each simulation iteration. These computed haptic forces are finally feedbacked to the device through the virtual surgical tool [12, 18–20, 27, 44, 53]. As a result, the user will feel like they are interacting with a real soft tissue when the reaction forces are received from the force-feedback device [3, 12]. For example, Cotin et al. used this combination in surgical simulation to provide haptic sensations for the surgeon [12]. Audette et al. used a 7-degree-of-freedom (DOF) haptic device combined with a surgical tool whose tip is fixed at the end of the haptic device to make the simulation system more realistic [18]. In the study of Chen et al., a commercial PHANTOM haptic device with 3 DOF force feedback and 6 DOF position and orientation was used for haptic surgery simulation [44]. Sela et al. developed a new force feedback system called the SensAble™ PHANTOM Desktop™ haptic device [20]. The device was combined with a pen-sized handle, and they are all connected to a robot arm with flexible engine-forced joints to simulate a virtual surgical scalpel. Courtecuisse et al. used a virtual laparoscopic grasper, which was managed by a Xitact IHP haptic device [27]. Peterlik et al. used a virtual haptic interface point (HIP) controlled by a PHANTOM haptic device to calculate haptic forces reacted from a liver model based on displacements between the HIP's position and the 3D surface model of the liver [3].

#### 3.6.1. 3D Scanners

The 3D scanners can be divided into two categories: structural and surface scanners. The most widely used structural scanners in the literature have been CT and MRI. Cotin et al. created an anatomical model of a human liver from MRI images [12]. Marchesseau et al. used CT images for creating the geometrical model of a liver [26]. Besides, 3D surface scanners were also used in the studies relating to surface-based soft-tissue modeling methods. The most popular surface-based scanners employed were laser scanners and ultrasonic scanners. They took advantage of measuring the time-of-flight of laser/ultrasound beams for estimating the distance between the laser/ultrasound sources and the object's surface. These scanners are fast and able to acquire the object surfaces in real time. The laser scanners are much more accurate than ultrasound scanners, but laser beams can be very harmful to the living soft tissues during long acquisition period. For example, Monserrat et al. employed the 3D ultrasonic scanner (SAC GP10, Smart EDDY System, USA) for capturing the 3D outside surface of the simulated object based on the boundary element modeling method [4]. The combination between surface scanners and structural scanners was also proven to be effective for accurate reconstructing both surface and structural details. Wang et al. combined a 3D laser scanner with the lateral X-ray scanners in their methods [48]. In fact, the 3D laser images containing both 3D geometrical point cloud and colors of the human face were transformed to the lateral X-ray image for comparing and cutting the nose part on the face. This combination provided a high-quality and patient-specific model of the human face appearance.

#### 3.6.2. Biomechanical Sensors

Most biomechanical sensors used in soft-tissue modeling systems are electromagnetic sensors, force sensors, and electromyography sensors. Brown et al. used an electromagnetic tracker (miniBRID of Ascension Technology Cooperation) to track behaviors of a real surgical forceps [2]. Sedef et al. attached a force sensor (ATI Industrial Automation's Nano 17) at the end of a real surgical probe for measuring forces inside a surgical trocar so that the user could feel like being in a real minimally invasive surgery [19]. Yarnitzky et al. employed ultra-thin force sensors arranged under the bony prominences of each foot for measuring the forces under the calcaneus, metatarsal heads, and phalanges in real time in an application of monitoring foot's internal deformations under outside interactions [58]. Electromyography (EMG) was also used in the study of Murai et al. for acquiring muscle tensions [5].

#### 3.6.3. The PC's Human Interface Devices

Some PC's human interface devices have also been used widely in the real-time soft-tissue simulation systems. They are all flexible and easy for user manipulation. For example, Lopez-Cano et al. used a PC mouse as a surgical tool interacted with a virtual pointer to deform the 3D model of the human inguinal region [45]. This configuration could simulate vertical and horizontal stretching deformations of the simulated model. In some simulated systems, the PC mouse could only be controlled to rotate the view angle of the simulated model [21]. Moreover, it could be used for drawing the cut shapes onto the surface model [48].

#### 3.6.4. 3D Viewers

The 3D viewers are the output interaction devices. They are composed of two separate high-resolution screens or two glasses attached together horizontally for displaying two different image frames to human eyes. Based on a stereo geometrical model from human vision, the 3D viewers can create depth perceptions for human brains, so when using the 3D viewer for visualizing the simulated model, the graphic rendering can become more realistic. A classic 3D viewer was presented by Brown et al. using the stereo glasses for enhancing the illusion of depth in the video frames [2]. In the visualization system, there were two image frames displayed: one image frame was colored in red, and the others was colored in cyan. The stereo glasses included two different color filter glasses for the left and right lens, so at the same time, each human eye would see a different image frame. Each pair of image frame was shifted horizontally for creating the depth information. A different 3D viewing device called a 3D stereo visor was used to visualize a simulated model in the virtual reality myringotomy simulation in the study of Ho et al. [6]. This device included two different high-resolution screens for displaying two different image frames at the same time.

#### 3.6.5. 2D and 3D Optical Cameras

The 2D optical cameras are the input interaction devices having the functions of acquiring 2D image frames of object surfaces. In the application of facial expression recognition, Chandrasiri et al. mounted a complementary metal oxide semiconductor (CMOS) camera to a headphone to capture 2D color video frames of a user face [42]. An ordinary web camera available on a mobile device was also used by Weng et al. in the application of real-time facial animations [52]. In particular, the offline 2D images acquired from a camera were also analyzed for detecting the facial expressions and cloning them to other 2D facial images in the study of Zhang et al. [51]. One of the most drawbacks of 2D optical cameras is the inability of reconstructing depth information from a single view of vision, so multiple optical cameras have been cooperated to form a 3D optical camera system for detecting the 3D data. A motion capture device was utilized to capture 3D motions of a human during dynamic movements in the study of Murai et al. [5]. A stereo optical motion capture was also combined with facial markers in the study of Wan et al. for detecting facial animations [49]. Over 36 facial markers were detected and followed by mocap, and their motions were then converted to MPEG-4 standard's definition of facial animations. Woodward et al. used an off-the-shelf stereo webcam for marker-based facial animation application [55]. Applied to minimally invasive surgeries in the study of Moroka et al., the 3D optical camera was integrated into a stereo endoscopy whose size was small enough to be used in restricted navigation spaces [31].

### 3.7. System Architectures

Computational approaches and interaction devices have been developed throughout the literature to improve computational accuracy and speed of soft-tissue deformation models, but they will not operate effectively and robustly in real time if soft-tissue models and interaction devices are not well-cooperated in a system architecture. This section will synthesize system execution schemes and programming frameworks of the system architectures developed in the literature.

#### 3.7.1. System Execution Schemes

Cotin et al. designed the first execution scheme called the distributed execution scheme in which a computer system and a Dec AlphaStation were closely cooperated [12]. The computer system is aimed at computing the haptic forces and exchanging data with the haptic device while the Dec AlphaStation visualized the deformations of this soft-tissue model in real time. The communication environment between two computer systems was the ethernet connection. Chen et al. distributed a developed haptic surgery simulation onto two computing systems [44]. While an SGI Prism Visualization Server with 4 ATI FireGL GPUs covered graphical simulations, a Windows computer system controlled the haptic devices and simulated the haptic feedbacks. The system could manage more than one simulated model by using a new peripheral protocol called virtual reality peripheral network (VRPN) developed by the University of North Carolina. Two workstation systems were also cooperated on a simulation system for the minimally invasive surgery proposed by Morooka et al. [31]. All model computations were performed by the first workstation while the second workstation only performs visualization of deformations and virtual tools in the form of a 3D stereo vision for improving depth sensations. Note that the limitation of transmission bandwidth leaded to the latency between the visualization force-feedback. To solve this issue, the multithread execution scheme was proposed, Brown et al., which distributed two tasks of deformation visualization and collision detection on two different execution threads on a single dual-processor machine (Sun Ultra 60 with two 450 MHz processors) [2]. The system included three intercooperative simulators: a deformable object simulator, a tool simulator, and a collision detection module. The idea of multiple-thread executing on a single computer system was also applied by Sedef et al. in a soft-tissue simulation system including a phantom haptic device, a computer screen, and a simulated model [19]. Peterlik et al. also implemented two asynchronous computation threads executed on an AMD Opteron Processor 250 (2 GHz) PC to operate a liver simulation system [3]. The main thread called haptic thread is acquiring positions of a haptic device, detecting collisions, calculating haptic forces, and computing model's deformations. The simulation system designed in the study of Goulette et al. also included multiple computation modules for accelerating the system execution [53]. Two modules were threaded to execute in parallel on an Intel Core 2 Duo at 2.40 GHz, 3.45 GB of RAM. As a result, the visual rate could be reached up to 47 FPS. Audette et al. designed a surgical simulation system which included their own developed haptic device with 7 DOFs [18]. To control this haptic device, an intelligent I/O board, called the DAP5216a/626, operating individually with the computer system was proposed. Another scheme for system execution called a multimodel representation, was proposed by Allard et al. within the SOFA framework [13]. In this scheme, each soft-tissue simulation components could be represented by multiple modeling methods related to real-time deformation simulation, accurate collision detection, or realistic interaction computation. Finally, the task of programmers was to design a switcher to effectively alternate modeling methods according to each appropriate simulation issue.

#### 3.7.2. System Frameworks

System development frameworks must be selected carefully so that the system could be developed both in high productivity and short time-to-market. Generally speaking, a software framework is a generalization software structure in which programmers can contribute their written codes to modify this structure to a specific application. Taking advantages of available configurations and prebuilt libraries, simulation systems could be developed much more flexibly and faster than in traditional development procedures. The system frameworks can be divided into four groups: the input/output interaction frameworks, the graphic interaction frameworks, the modelling frameworks, and the hybrid frameworks. Regarding the input/output interaction frameworks, haptic devices have been commonly used in the literature, and they are often interfaced with computer systems by GHOST [3, 19, 44] and PHANTOM [44] input interaction framework. These input interaction frameworks are all free and open source. Moreover, other standard input interaction devices, such as keyboards, PC mouse, web cameras, and microphones, can also interface with computer systems through application programming interfaces (APIs) supported by Microsoft Windows systems [42, 45, 48]. Regarding the graphic interaction frameworks, the most employed graphic framework was OpenGL in which 2D and 3D vector graphics can be rapidly rendered by GPU-platform boards. The rendering tasks can be executed on a separate computer system or on a local thread [3, 6, 18, 19, 38, 44, 45, 59]. In particular, the OpenGL framework can be embedded in multiple types of operating systems such as Android, iOS, Linux, Windows, and various embedded operating systems. Moreover, it can also support for writing in multiple programming languages (e.g., C++, Python, C#, and Cg). In addition, the CUDA™ graphic framework was developed by Nvidia Corporation. There have been numerous studies using CUDA framework for implementing their simulation system on of-the-shelf graphic GPU boards and achieving great benefits from parallel execution structure in real-time computations [17, 23, 24, 27, 32, 34]. Another general graphic framework called OpenCL™ was also developed for flexible parallel implementation. An image processing framework called Virtual Place (AZE Co.) was also used for converting 3D deformations to stereo video frames for creating depth feeling on human visions [31]. Regarding the modelling frameworks, GHS3D [26], TetGen [47], and CDAJ-Modeler [31] were employed for generating mesh models from CT/MRI images. Additionally, Maxilim software could also support for boundary condition simulations [43]. Moreover, the CHOLMOD open source library could also be used for solving the linear systems in real time [50]. Finally, the combinational frameworks have been developed to provide a much more flexible and multifunctional environment for developing a whole system. MATLAB is a powerful combinational framework including a facial analysis toolbox used for facial expression analysis [42]; a toolbox called iso2mesh was used to generate a tetrahedral mesh of a brain and its tumor [7]; an artificial neuro network toolbox was employed to train the force-deformation data [7]; an optimization toolbox was used to obtain optimal parameters of simulation models that represent for simulated physical quantities; the OpenGL graphic library could be supported in the MATLAB environment for simulating interaction between soft-tissue model and surgical tools [47]. An Android programming platform was also used [52]. Additionally, supporting for FEM physical modelling, the Fast FE Modelling Software Platform [14] and GetFEM++ [3] were also employed. For parallel threading, the RTAI-patched Linux was used for satisfying the hard real-time requirements [18]. Other powerful and more multifunctional system frameworks are the SOFA [13, 26, 29, 33] and CHAI3D [6] frameworks. In fact, they support various libraries and modules for implementing a complete simulation system including input/output interaction device drivers, geometrical model libraries, modelling algorithms libraries, and graphic rendering modules.

### 3.8. Clinical Validations

To translate the outcomes of the developed simulation systems into clinical routine practices, a systematic validation must be required. All validation efforts done in the literature were ordered as three validation levels: geometrical validations, model behavior validations, and user acceptability/safety validations.

The most important components in a simulation system are the geometrical and physical models. To accurately simulate the target soft tissues, the geometrical appearances of both the simulated models and real objects must be well-fitted. Geometries at a specific state are commonly compared with the real *in vivo/in vitro* data acquired from a relatively accurate measurement method. CT/MRI were usually used to reconstruct the real 3D geometrical models of the tissues of interest. Due to expensive processing time and resources, this scheme is just suitable for offline geometrical validation. For example, real data from patients under maxillofacial surgeries were stored and compared with the predicting appearances for improving the reproducibility capacity of the simulation system [43]. Moreover, the soft-tissue phantom could be used to give the validating data for the geometrical validation [7]. Regarding the model behavior validations, the physical characteristics of the simulated models must be assessed with the real physical data at different deforming states. One of the most popular schemes is to use the calculated data from a standard commercial simulation software as baseline data. For example, a model using linear viscoelastic FEM was validated through a compression test solved by both the proposed computation approach and the ANSYS finite element software package. Obtained results showed that the maximum error of displacement was less than 1% [19]. The ANASYS software package was also used for validating a model based on a machine learning-based FEM method [36]. Recently, the Marquardt-based model has also been validated by ANSYS in a liver simulation system [56]. The Abaqus software was also used for validation purpose [17, 24, 32]. Other used FE packages relate to MSC NASTRAN 2003 which was used by Yarnitzky et al. [58] and LS-DYNA™ which was used by Joldes et al. [15]. Note that open source packages were also employed. The SOFA framework was the execution environment for performance evaluation between the developed method and the previous modelling methods [26]. In addition to geometrical and model validations, user acceptability/safety validation needs to be performed to evaluate the quality of interfaces between the system and its users in real clinical applications. One of the most popular schemes of this validation level is to collect feedbacks from experts and patients who have been experienced with the developed system. Ho et al. validated their virtual reality myringotomy simulation system by a face-validity study in which a validated questionnaire was delivered to eight otolaryngologists and four senior otolaryngology residents for evaluating the system after a long period interaction with the simulator [6]. Tonutti et al. conducted their validation procedure on surgeons with and without implementing the developed system, and the difference results were evaluated for proving the effectiveness of the simulation system [7].

## 4. Discussion

### 4.1. Computational Approaches

The FE modelling methods and its variations have been commonly used for developing soft-tissue deformation models. However, the trade-off between system accuracy and computation speed remains a challenging issue. The FEM can simulate deformations for complex soft tissues [67, 68]. In particular, a commercial FE solver was commonly used to evaluate the accuracy of a new FE algorithm [21, 22, 24, 32]. However, using the FE method, real-time requirements are only satisfied if being modelled with a smaller number of elements [30, 35] or being accelerated by GPU implementation [23, 24]. In general, the computational cost of the FEM increases exponentially with the expansion of the number of nodes especially in case of simulation of the nonlinear materials. Consequently, various development techniques have been developed to improve their computation speeds. The most used approach was the precomputation-based technique. This approach has been proven as a fast and robust technique for simulating deformations in real time, but large deformations with complex material properties and constitutive laws and topological changes on the fly could not be handled during the system iterations because the trained model cannot be updated online [3, 7, 8, 12, 19, 20, 31, 36, 37]. Other FEM variations could significantly improve the performance of the FEM. One of them was the case of linearizing the kinematic of the simulated object [14] in which the model could be cut faster than the original model using FEM, but the speed was not fast enough for realistic visualization in medical applications. The idea of dividing a FEM mesh into multiple submeshes to be executed in parallel [18] could initially increase the computation speeds, but this method leaded to the limited number of threads being able to handle on a real-time operation system. Other development methods such as matrix system reduction (MSR-FEM) [21] and the order reduction method (ORM-FEM) [29] based on the reduction of the FEM's stiffness matrix could improve the processing time, but they just simulated small deformations. The total Lagrangian algorithm in conjunction with FEM [22] and its modification known as total Lagrangian explicit dynamic (TLED-FEM) [16] allowed element precomputations, so a less computation cost would be required for each time step. It is important to note that hyperelastic and viscoeleastic constitutive models were implemented with explicit time integration schemes in a straightforward manner using homemade or commercial FE solvers. Moreover, multiplicative Jacobian Energy Decomposition (MJED-FEM) [26] with implicit time integration schemes could be used to model hyperelastic, viscoelastic, and poroelastic behaviors of the soft tissues. However, these methods could not handle interactions with other simulated objects and topological changes. The topological changes could be handled in the method proposed by Turkiyyard et al. [28], but they could not solve effectively the cured cuts, partial cuts, and multiple cuts inside elements. More effectively for simulating the topological changes was element-by-element precondition conjugate gradient FEM (EbE PCG-FEM) [32] method, but it was not suitable for simulating the heterogeneous materials. Another potential method called preconditioning FEM (pre-cond FEM) [33] could solve this problem dramatically. It could both simulate the topological changes and the haptic feedback of homogeneous and heterogeneous materials with acceptable accuracy and real-time frame rates.

On the other hand, the meshfree-based techniques have been achieved great attention in the recent years. All the meshfree-based methods have been very fast and highly adaptive to topological changes, but they are less realistic than the mesh-based methods from biomechanical point of view. The most popular meshfree-based method was mass-spring system (MSM) [2, 6, 38, 44, 45]. This method could handle the deformations and topological changes, but it could not simulate accurately with nonlinear material characteristics. The improvements of MSM method were the mass-tensor method (MTM) [43] and mass-spring-damper (MSD) method [47]. They could handle the nonlinear material more effectively than the MSM due to the use of nonlinear mass springs in the method. Another improvement of MSM was the hyperelastic mass link (HEML) method [53]. This method could be considered for the compromising solution between the biomechanical accuracy and computation efficiency in real time. A different technique was point collocation-based method of finite sphere (PCMFS) [46]. By just focusing on the local region of interests, the simulation time could be decreased significantly, but the method for detecting the region of interest was still not defined effectively. More globally, the method called inverse dynamic computation (IDC) [5] could compute the muscle tensions based on the external data from sensors. Despite of the acceptable accuracy, the method could not analyze a single muscle. This idea could be found in the method called elastic-plus-muscle-distribution-based (E+MD) [51] in which the facial expression could be used for investigating the internal muscle tensions. A recent method called time-saving volume-energy-conserved ChainMail (TSVE-ChainMail) [54] was powerful in handling both topological changes and simulating the isotropic, anisotropic, and heterogeneous materials at the real-time rate. At this stage, the real-time deformations could be achieved but the interactions among the simulated objects remains a difficult task. To overcome this drawback surface-based methods (e.g., boundary element method (BEM) [4], Laplacian surface deformation (LSD) [41], and Marquardt radial basis meshless method (MRM) [56]) have been proposed. These approaches could estimate the internal deformations based on the surface changes, and it could handle the interaction between modelled soft tissues through surface interactions, but they were not able to simulate inhomogeneous materials, nonlinear elastics, and topological changes on the fly. In addition, model cutting and needle penetration issues were also studied using the extended finite element method (XFEM) and meshfree-based approaches for soft tissues. In particular, the extended finite element method (XFEM) has been used to study complex hard tissue (tooth [69], maxillary molar, and endodontic cavities [70]) models with fracture and crack propagation behaviors and soft tissue (cornea [71]) models with cutting simulation. This open new avenue to model biological tissues with more complex interaction behaviors.

In addition, many studies have been conducted for the implemented model based on developed soft-tissue deformation method on to the GPU-parallel computing platform, and they can all achieve much better accelerations when compared with the conventional developing approach. Not all developed modeling methods are suitable for this approach, so the implicit time integration of nonlinear FEM method has been proven to be the most suitable for parallel implementation. Furthermore, the additional reconfigurations must be approved to the current methods to adapt with the implemented hardware platforms. When the model developing approach reaches its limitation, new implementation strategies will be necessary for accelerating their current computation performances.

It is important to note that the computation speed and resources depend on each particular application (e.g., surgical planning or surgical simulation) of soft-tissue deformation systems. For example, real-time soft-tissue deformation behavior, high-speed device interaction, and skill-based training ability could be more important criteria to be achieved for a computer-aided surgical simulation system. Besides, surgical planning system focuses on the whole workflow from data acquisition and previsualization of a specific surgical intervention and then predefine the optimal surgical steps.

Generally speaking, a large range of methods were developed to simulate the soft-tissue deformations. Each method showed its robustness and accuracy for a specific case study. There exists no universal methods, and the selection of the methods depends directly on the application. It is important to note that real-time deformations with topological changes on the fly, and accurate object interactions remain challenging issues. One of the potential solutions relates to the use of multiple modeling methods in a whole simulation system. However, an effective cooperation strategy should be established, and the requirement of advanced computational resources needs to be satisfied.

Finally, the computation speed of a soft-tissue simulation system depends strongly on the use of constitutive behavior laws for modeling soft-tissue physiology. Elastic, hyperelastic, and viscoelastic laws were commonly used in the developed real-time simulation systems for the upper/lower limb muscle, facial muscle, liver, and skin tissues. It is important to note that more complex constitutive laws such as electromechanical models could be used in general for modeling the skeletal muscle [68] or myocardium [72, 73]. However, these complex models deal with additional computational need and requirements to reach a real-time ability for medical simulation systems. Linear and nonlinear stress-strain relationships were described in the elastic material. Hyperelastic material was described using Neo-Hookean and Mooney-Rivlin formulations. It is important to note that some additional components were integrated into linear elastic law to improve the computation speed and model accuracy. For example, the combination of a linear elastic law with a corotational method was performed (Courtecuisse et al. [33]) or an extra mass-spring model was integrated into a linear elastic law (Zhu and Gu [59]). Regarding all analyzed simulation systems for soft tissues, the most used law is the linear elastic one. The use of more complex laws (hyperelastic and viscoelastic) leads to a larger number of model parameters and of course computation speed.

### 4.2. Interaction Devices in Real-Time Simulation Systems

There have been various kinds of interaction devices contributing differently to the system's reality and computation performance. While the output interaction devices mainly provide realistic visualizations and reactions to human senses, the input interactions have the fundamental involvement to the computation performance, especially in both model accuracy and computation speed. Regarding the output interaction devices, the computer screens display appearances of simulated models and their deformations when interacted with virtual surgical instruments and/or other surrounding structures [6]. However, their lacking of depth information makes visualizations possible only in 2D space. The 3D viewers can complement this drawback. Like human visions, this interaction devices can create 3D virtual sensation for human vision based on the differences between left and right scenes [2, 6]. Even more realistically, the haptic feedback devices receive calculated haptic forces from simulated models to create collision feeling for human tactile [3, 12, 18, 20, 27, 44, 53, 57]. Consequently, the cooperation between the 3D viewers and the haptic feedback devices will become much more powerful in generating realistic sensations for humans [2, 6]. In particular, force-feedback devices have been commonly used for many medical applications (e.g., surgical simulation, surgical trainings, or minimally invasive surgeries [18, 44, 53]). Force-feedback devices have been flexibly cooperated with various types of virtual surgical tools (e.g., virtual haptic interface point (HIP), the virtual blade, or the virtual scalpel). The most widely haptic device used in the literature is the SensAble™ PHANTOM Desktop™ haptic device, and its flexibilities are dependent on the number of DOFs. It is important to note that to simulate force feedbacks realistically, the haptic forces must be estimated and transferred to the force-feedback device at speeds from 500 Hz to 1000 Hz, so this means that the computation speeds of simulated models must be faster than those speeds [18, 57]. Furthermore, a separate controller must be installed and executed one or multiple computer system to keep the real-time computation speed [3, 19, 27, 44, 53].

On the other hand, several biomechanical quantities have not been measured directly from the biomechanical sensors, so simulated models are often used to infer internal physical characteristics based on external knowledge. For example, in the case of musculoskeletal tracking, EMG sensors are often fused with musculoskeletal models for inferring individual muscle tensions according to the markers' motions, which are tracked by the 3D optical camera system [5, 58]. The soft-tissue physical parameters can also be inferred from soft-tissue deformation models by the movements of surface markers instead of direct measurements from the sensors. The surface makers are proven to be very robust and flexible for estimating outside deformations, but the limited number of markers being able to put on a soft-tissue surface leads to decrease the estimated deformation resolutions and so are the resultant calculations [31, 49, 55]. The 3D scanners such as MRI/CT scanners [8, 12, 26] and 3D ultrasonic scanners [4] have been employed for detail surface reconstruction in 3D spaces, but their slow acquisition times (in case of MRI/CT scanners), lacking of surface characteristics (in case of 3D laser scanners and ultrasonic scanners), and harmful infections to human health (in case of CT and laser scanners) make them not suitable for tracking external deformations of soft tissue in real time and in long-term use. This issue was initially resolved by the combination between the 2D optical cameras and the X-ray images for adding more surface characteristics [48], but the appearances were static and could not estimate deformations on the fly. Consequently, other devices having the ability of acquiring both detail surface deformations in 3D spaces and surface characteristics online are substantially required for improving computation speeds and model accuracy of soft-tissue simulation systems.

### 4.3. Suitable Execution Scheme in Simulation Systems

To manage the data transmission from/to I/O interaction devices and to compute the simulated model in an optimal way, a suitable system execution scheme must be developed. There are two main system execution schemes. In the distribution-based scheme [12, 31, 44], system tasks are highly parallelized in multiple computing machines, which are interfaced through a limited bandwidth and slow-transmission environments. This scheme allows system tasks to execute independently and take advantages of multiple computing hardware, but the problems appear when having delays in communication between multiple machines. Thus, data transmissions are still not fast enough for effectively communicating among multiple computer systems. This issue has been initially solved by numerous attempts such as high-speed ethernets and high-speed data transmission protocols, but they are not efficient enough for transmitting large information in real-time. In the multithread scheme [2, 3, 19, 53], system tasks are executed on multiple computing threads. Because all threads are connected through a very high-speed internal bus, there is nearly a delay in data transmission among threads. However, because of the limitation of computation strength and memory capacity of a single thread inside a computer system, the simulation task(s) must be simplified and optimized to be able to execute on a single thread. This can be a challenging task for model developments and implementations. Fortunately, this challenge can be easily resolved by the development of hardware technology with more threads integrated on a single CPU or even more CPU facilitated on a single computer system. In addition, cooperation of the two execution schemes was also found in the literature [13, 18]. In this case, various types of data acquisition boards have been developed to fast manage the input/output data streams. These boards are designed in a mobile hardware and can be easily plugged in to a computing machine through a specific high-speed transmission channel and a software driver. Consequently, this system configuration can take advantages of both distribution-based and multithread-based execution schemes.

### 4.4. Clinical Validations

Generally speaking, the clinical validation is the final system development stage to determine whether the simulation system is acceptably suitable for clinical routine practices. Current clinical validation procedures were grouped into three levels: geometrical validations, model behavior validations, and user acceptability/safety validations. Regarding geometrical and model behavior validations, the validation data have been commonly acquired from standard simulation software, phantom soft-tissue organs, or postoperation data. There is a lack of *in vivo* data for accurately validating the simulation system in real medical environments. The use of accurate CT/MRI data is promising, but this approach is not suitable for online validation. The use of standard simulation software to validate the physical behaviors of simulated models also faces some problems. It is important to note that most of soft-tissue materials (e.g., muscle, fat, and skin) are unavailable in these types of software. Thus, only simplified behaviors were validated with classical mechanical laws (e.g., linear elastic or hyperelastic laws). Consequently, more experimental protocols should be investigated to characterize the soft-tissue behaviors and use them for enhancing model behavior validations. Finally, the user acceptability/safety validation was performed with the end users including patients, trainees, and experts through questionnaires. Note that this approach is relatively subjective and qualitative. In addition to these validation levels, system validation should be performed in which the whole system was evaluated rather than each system's components. This stage targets at analyzing system functions, system robustness, and system computation performances during short-term and/or long-term working durations. While the system functions are relatively easy to verify by comparing with the proposed development functions at the designing stage, the system robustness and computation performances must be tested after short-term and long-term working durations. Although this validation process is necessary for a stable and robust system, rarely, studies in the literature conduct this validation.

## 5. Current Trends, Limitations and Future Recommendations

The trends of the current computational approaches relate to (1) mathematical formulation of physical laws applicable on image-based soft tissue geometries, (2) real-time simulation achievement of soft-tissue deformation with simple constitutive laws, and (3) model implementation on specific hardware configuration to speed up the computational time. However, soft-tissue behavior is commonly anisotropic, viscoelastic, inhomogeneous, and nearly incompressible with large deformation. In fact, the consideration of all physiological aspects is practically difficult, particularly for a real-time simulation system. Thus, modeling assumptions related to constitutive laws, geometrical discretization, and boundary and loading conditions were commonly performed for a specific application. Further studies need to be investigated to develop more accurate computational approaches for simulating complex soft-tissue behaviors in real-time conditions. The hybrid modelling approach in combining several methods is a potential solution leading to maximize the advantage of each method and overcome the limitations of the other.

Concerning the interaction devices, the ability of acquiring multiple types of data both in real-time and accurate manner and the portability of sensors are the current trends. Multiple sensors could be embedded into a single well-calibrated structure and worked as an independent configuration. These types of sensors, therefore, are more accurate and faster than manually calibrated sensor systems. In fact, some multiple function sensors such as the KINECT™ developed by Microsoft®, XTION™ developed by Asus, and other well calibrated stereo cameras are good recommendations for this requirement. However, current sensors are difficult to acquire deep information on the soft tissues, which are crucial for *in vivo* modeling and simulation. In particular, there have been no sensors having the ability of acquiring these data in real time, so there is a need for a new type of sensor that can get the internal structures and/or textures in real time. In fact, complex data processing schemes need to be investigated in the future to study the external-internal relationship of the soft tissues leading to a predictive solution of internal structures from external information. Statistical shape modeling (SSM) or artificial intelligence- (AI-) based approaches are potential methods for such complex objective.

Regarding the system architecture and execution scheme, the availability of powerful and open frameworks for medical imaging processing (e.g., 3D Slicer), data visualization (e.g., OpenGL, VTK), and simulation (e.g. SOFA) is the current trend, which can speed up the development of new systems. However, the compatibility between these frameworks becomes a potential drawback. To deal with this obstacle, the community should work together to define a common computational protocol and promote its use within any system development for future applications. In addition, all developed system execution schemes are very hard to program without the help of system frameworks. Most used system frameworks mainly supported for programming multithread schemes rather than distributed schemes and combination schemes. Moreover, they did not well manage the memories between internal threads. For further recommendations, more system frameworks should be developed for supporting the communication between threads. Furthermore, frameworks for programming, the distributed schemes also need to be investigated for supporting connection and data transmission between multiple computing machines. More software development kits (SDKs) should be developed for general sensors such as single cameras, stereo cameras, and laser scanners so that deeper information could be extracted and estimated. In addition, to translate developed systems into clinical routine practices, software development workflow dedicated for medical application should be followed to ensure a high-quality and reliable medical software for the benefit of involved patients and clinicians.

Finally, multilevel validation becomes an avoidable task when developing a real-time medical simulation with soft-tissue deformation. Quantitative assessment of accuracy is also focused. However, when developing a soft-tissue simulation system, a systematic validation procedure must be simultaneously proposed. Specifically, a system must be sequentially validated through all validation levels. The type of validating data for each validation process should be clearly defined, and acquisition processes should also be planned. Moreover, an assessment program could be included in the system to evaluate the development progress of the users during each level of trainings. In user acceptability/safety validations, a supervising task programmed to run simultaneously with the simulation system could be used to track users' behaviors. Additionally, a function that include questionnaires for evaluating user acceptability could be included in the system's functions, so the results are automatically processed and sent to developers for further development.

## 6. Conclusion

The present review paper was conducted to summarize the literature works about real-time soft-tissue deformation systems. Throughout this review, studies relating to real-time soft-tissue simulations have been analysed according to four system engineering aspects: computational approaches, interaction devices, system architectures, and clinical validations. This review provides useful information to describe how each aspect has been developed and how they have been cooperated for both executing in real time and keeping realistic behaviors of soft tissues. By clearly analysing advantages and drawbacks in each system development aspect, this review paper can be used as a reference guideline for system developers to choose their suitable system's components while developing soft-tissue simulation systems. Finally, this review paper identified some recommendations for future researches.

## Figures and Tables

**Table 1:** The search terms used for the systematic review process.

#	Search terminologies (terms)	Search terms (STs)
1	Term #1: Computer-aided medical simulations/systems	ST #1: Real-time AND computer-aided AND medical AND (simulations OR systems)
2	Term #2: Real-time biomedical simulations/systems	ST #2: Real-time AND biomedical AND (simulations OR systems)
3	Term #3: Real-time facial simulations	ST #3: Real-time AND facial AND simulations
4	Term #4: Real-time liver deformation models	ST #4: Real-time AND liver AND deformation AND models
5	Term #5: Real-time medical simulations/systems	ST #5: Real-time AND medical AND (simulations OR systems)
6	Term #6: Real-time muscle deformation models	ST #6: Real-time AND muscle AND deformation AND models
7	Term #7: Real-time surgery	ST #7: Real-time AND surgery
8	Term #8: Real-time finite element methods	ST #8: Real-time AND finite AND element AND methods
9	Term #9: Real-time soft-tissue deformations	ST #9: Real-time AND soft AND tissue AND deformations

**Table 2:** The number of included/excluded articles according to the selection procedure.

Search terms	ScienceDirect	PubMed	IEEE	All	Duplicates	Duplication included	Title excluded	Title included	Abstract excluded	Abstract included	Content excluded	Content included
ST #1	5,537	67	13	5,617	21	5,596	5,465	131	128	3	2	1
ST #2	10,873	2,873	284	14,030	447	13,583	13,542	41	37	4	3	1
ST #3	3,638	87	14	533	19	514	335	179	169	10	0	10
ST #4	1,689	39	10	1,738	19	1,719	1,543	176	171	5	4	1
ST #5	32,857	9,762	290	42,909	605	42,304	42,209	95	83	12	7	5
ST #6	4,560	21	3	4,583	19	4,564	4,431	133	125	8	5	3
ST #7	104,034	31,339	367	135,727	371	135,356	135,265	91	76	15	3	12
ST #8	146,837	261	153	147,251	60	147,191	147,136	55	45	10	7	3
ST #9	9,185	160	23	9,368	49	9,319	9,247	72	47	25	6	19
Total	319,210	44,609	1,157	361,756	1,610	360,146	359,173	973	881	92	37	55

**Table 3:** The inclusion criteria for each search terminology.

**#**	Search terms (STs)	Inclusion conditions (ICs)
1	ST #1	IC #1.1: the title must satisfy all of the following conditions: (1) the title contains “real-time”, “medical”, “simulations”, and “computer-aided” keywords and (2) the title concerns the supports of computers in soft-tissue simulations executing in real time
		IC #1.2: the abstract must satisfy all of the following conditions: (1) the abstract concerns the support of computer in medical systems, medical simulations, and medical applications so that they can be executed in real time; (2) the abstract describes the medical system architectures and the interactions of computer's input/output devices in clinical environments; and (3) the system developed in the paper focuses on simulating human soft tissues

2	ST #2	IC #2.1: the title must satisfy all of the following conditions: (1) the title contains “real-time”, “biomedical”, and “simulations” keywords and (2) the title concerns the issues of real-time simulation in biomedical applications
		IC #2.2: the abstract must satisfy all of the following conditions: (1) the abstract concerns the analyses of real time in biomedical applications/systems and (2) the abstract focuses on analysing the computational approaches, the system architectures, or the characteristics of real time in biomedical applications

3	ST #3	IC #3.1: the title must satisfy all of the following conditions: (1) the title contains “real-time” and “facial” keywords, and (2) the title concerns the computational approaches to simulate the human faces
		IC #3.2: the abstract must satisfy all of the following conditions: (1) the abstract concerns the development of computational techniques or system designs for modelling the facial mimics/expressions/muscles and (2) the developed techniques must be able to execute in real time

4	ST #4	IC #4.1: the title must satisfy all following conditions: (1) the title contains “real-time”, “liver”, and “models” keywords and (2) the title concerns the modelling methods of the human liver in real time
		IC #4.2: the abstract must satisfy all following conditions: (1) the abstract concerns the issues of computational approaches for modelling the human liver and (2) the computational approaches must be executed in real time

5	ST #5	IC #5.1: the title must satisfy all of the following conditions: (1) the title contains “real-time”, “medical”, and “simulations”/”systems” keywords and (2) the title is aimed at developing the computational methods for modelling the soft tissue in medical environments
		IC #5.2: the abstract must satisfy all of the following conditions: (1) the abstract concerns computational approaches or system architectures for modelling soft tissues in medical environments and (2) the system must be run in real time

6	ST #6	IC #6.1: the title must satisfy all of the following conditions: (1) the title contains “real-time”, “muscle”, and “models” keywords and (2) the title considers the computational methods for modelling the human muscles in real time
		IC #6.2: the abstract must satisfy all of the following conditions: (1) the abstract concerns the developments of computational techniques for modelling and simulating human muscles so that they can run in real time and (2) the abstract shows the implementations of muscle deformable models in clinical environments

7	ST #7	IC #7.1: the title must satisfy all of the following conditions: (1) the title contains “real-time” and “surgery” keywords and (2) the title illustrates the surgical simulations/systems applied in human soft tissues executed in real time
		IC #7.2: the abstract must satisfy all of the following conditions: (1) the abstract describes the surgical simulations/systems for human soft tissues and (2) the abstract concerns system architectures of surgical simulations or systems so that they can execute in real time

8	ST #8	IC#8.1: the title must satisfy all of the following conditions: (1) the title contains “real-time” and “finite element” keywords and (2) the title concerns the finite element modelling methods for human soft tissues in real time
		IC #8.2: the abstract must satisfy all of the following conditions: (1) the abstract concerns the human soft-tissue modelling method in real time based on the finite element modelling methods and (2) the abstract is aimed at developing, generating, and analysing the variations of finite element modelling methods to get the real-time requirements

9	ST #9	IC #9.1: the title must satisfy all following conditions: (1) the title contains “real-time”, “soft tissue”, and “deformations”/”models” keywords and (2) the title considers the modelling methods of the human soft-tissue deformations executing in real time
		IC #9.2: the abstract must satisfy all of the following conditions: (1) the abstract illustrates the computational approaches for development the models of human soft-tissue deformations and (2) the abstract is aimed at developing, analysing, and generating the modelling methods

**Table 4:** Summary of the statistical results of the quality assessment procedure.

Quality assessment criteria	% of “yes” scores (%)
Computational approaches' bias	
1. Was the method adequately used/developed and described for the involved tissue behavior?	82
2. Was the verification well performed for the used/developed method?	76
3. Was the validation systematically performed for the used/developed method?	89
4. Did the method really satisfy the real-time constraints?	65
Interaction devices' bias	
5. Was the devices well selected for the system?	49
6. Was the device accuracy adequate for the real-time constraint?	47
7. Was the device easy enough to use for a clinical routine practice?	47
8. Is the device price suitable for a clinical setting?	47
System architectures' bias	
9. Was the system adequately described?	65
10. Was the system developed with the participation of the end users?	15
11. Was the system scalable?	53
12. Were the system frameworks adequately selected for implementing the system of interest?	45
Clinical applications bias	
13. Was the study adequately validated with *in vitro* data?	33
14. Was the study adequately validated with *in vivo* data?	13
15. Was the study adequately validated with patient data?	18
16. Was the level of validation suitable for translating the outcomes into clinical routine practices?	29
17. Was the user acceptability performed for patients?	4
18. Was the user acceptability performed for clinical experts?	7

**Table 5:** Classification of developed modelling methods for soft-tissue deformations in real time: mesh-based techniques.

Reference	Approach	Modelling methods	Soft-tissue types	Tissue behaviors	Computation time/speed	Geometry discretization	Hardware configurations
Cotin et al. [12]	MD	Precomputation-based FEM (pre-comp FEM) approximated by linear functions	The human liver	Linear elasticity Nonlinear elasticity	7 ms (force feedback) 8 ms (force feedback)	1400 N^∗^ 6,500 tetrahedral elements	Dec AlphaStation 400 MHz

Berkley et al. [14]	MD	Linearized FEM (L-FEM)	The human skin	Linear elasticity	1 kHz (force feedback) 30 Hz (model rendering)	863 N Surface triangle elements	1 GHz Athelon CPU

Audette et al. [18]	MI	Multirate FEM (MR-FEM)	The human brain	Linear elasticity	10 kHz (force feedback)	NI^∗∗^	Dual Pentium PC

Sedef et al. [19]	MD	Precomputation-based FEM (pre-comp FEM) using linear viscoelastic formulations	The soft-tissue cube	Linear viscoelasticity	1 kHz (force feedback) 100 Hz (model rendering)	51 N 153 DOF^∗∗∗^ 136 tetrahedral elements	Pentium IV 2.4 GHz dual CPU

Sela et al. [20]	MD	Precomputation-based FEM (pre-comp FEM) using discontinuous free form deformations	The human skin	Linear elasticity	1 kHz (force feedback and model cutting)	12,108 polygons	P4-2.8 GHz CPU, 1 GB RAM

Karol Miller et al. [16]	MD	Total Lagrangian explicit dynamic (TLED) FEM	NI	Nonlinear elasticity	16 ms (model deformation)	6000 E^∗∗∗∗^, 6741 N Hexahedral elements	3.2 GHz Pentium IV

García et al. [21]	MD	Matrix system reduction FEM (MSR-FEM)	NI	Linear elasticity	3.8 ms–35.7 ms (solving the system)	From 266 N–1,579 E to 110 N–587 E	2.4 GHz Pentium IV CPU, 1 GB

Joldes et al. [22]	MD	Total Lagrangian (TL) FEM	NI	Nonlinear elasticity	2.1 ms (one system time step)	2,200 E-2535 N Hexahedral elements	CPU

Taylor et al. [17]	MI	Total Lagrangian explicit dynamic (TLED) FEM	The human brain	Nonlinear elasticity	From 14.0 to 10.7 times faster than CPU	From 11,168 E to 46,655 E Tetrahedral elements	3.2 GHz P4 CPU, 2 GB RAM NVIDIA GeForce 7900 GT GPU

Joldes et al. [15]	MD	Total Lagrangian explicit dynamic FEM (TLED-FEM)	The human brain	Hyperelasticity (neo-Hookean)	12 ms (model deformation) 1 kHz (haptic feedback)	15,050 E, 16,710 N 7,000 DOF	3 GHz Intel Core Duo CPU

Joldes et al. [23]	MI	FEM (NL-FEM) implemented on GPU	The human brain	Nonlinear elasticity	3.54 s (3000 system time step running) 19.95 s (3000 system time-step running)	16,825 E-12,693 N 125,292 E-95,669 N	GPU NVIDIA CUDA Tesla C1060 (240 1.296 GHz cores, 4 GB high-speed memory)

Wittek et al. [24]	MI	Total Lagrangian explicit dynamic FEM (TLED-FEM) implemented on GPU	The human brain	Nonlinear elasticity	<4 s (deformation prediction)	18,000 N–30,000 E ~50,000 DOF	GPU NVIDIA CUDA tesla C870 (128 600 MHz cores, 1.5 GB memory)

Peterlík et al. [3]	MD	Precomputation-based FEM (pre-comp FEM) using radial basic functions (RBF)	The human liver	Nonlinear elasticity	0.54 s 9.89 s (stiffness and tangent stiffness matrix computing) 1 kHz (haptic feedback) 30 Hz (model rendering)	1,777 E–501 N 10,270 E–2,011 N Surface triangle elements	AMD Opteron 2 GHz CPU, 8 GB RAM

Lapeer et al. [25]	MI	Total Lagrangian FEM (TL-FEM)	The human skin	Hyperelasticity (general polynomial, reduced polynomial, and ogden formulation)	>1 kHz (haptic feedback)	100 E–50,000 E	GPU

Marchesseau et al. [26]	MD	Multiplicative Jacobian energy decomposition FEM (MJED-FEM)	The human liver	Porohyperelasticity, Viscohyperelasticity	13 FPS (model deformation)	20,700 E–4,300 N Tetrahedral elements	CPU

Courtecuisse et al. [27]	MI	Linearized FEM (L-FEM)	The human cataract The human liver The brain tumor	Linear elasticity combined with a corotational method	1.4 FPS (model computing model on CPU) 46.15 FPS (model computing on GPU) 64 ms (model computing on GPU)	41,000 N Tetrahedral elements 3,874 N Tetrahedral elements	GPU

Turkiyyah et al. [28]	MD	Discontinuous basic function FEM (DBF-FEM)	The human skin	Linear elasticity	13.9 ms (model computing and mesh updating)	31,008 N Surface triangle elements	CPU

Niroomandi et al. [29]	MD	Order reduction method (ORM) FEM	The human cornea The human liver	Nonlinear elasticity	20 Hz (model and graphic updating)	7,182 E–8,514 N Hexahedral elements 10,519 E-2853 N Tetrahedral elements	2 GHz CPU, 2 GB RAM

Wu et al. [30]	MD	Finite element method (FEM)	The superficial fascia in a face	Nonlinear elasticity	NI	560 E–1180 N 28,320 DOF	CPU

Morooka et al. [31]	MD	Precomputation-based FEM (pre-comp FEM) using neuro networks	The phantom liver	NI	NI	15,616 E-4,804 N	CPU

Mafi and Sirouspour [32]	MI	Element-by-element precondition conjugate gradient FEM (EbE PCG-FEM)	The human stomach	Linear elasticity	10 times faster than CPU for model computing	6361 E–13,3784 E 1295 N–25462 E	NDIVIDA GTX 470

Courtecuisse et al. [33]	MI	Precondition FEM (pre-cond FEM)	The heterogeneous soft tissues	Linear elasticity combined with a corotational method	70 FPS (system iteration) 1 kHz (haptic feedback) 22 ms (node adding or removing)	1,300 tetrahedral elements 150 contact points 3,874 N	256 core GPU

Strbac et al. [34]	MI	Total Lagrangian explicit dynamic (TLED) FEM	A general cube mesh	Hyperelasticity (neo-Hookean)	0.309 s–163.402 s (one solution time step)	125 E–91,125 E	NVIDIA GTX460 GPU

Karami et al. [35]	MD	Finite element modelling method (FEM)	The extraocular muscles (EOMs) in an eye	Linear elasticity	20 ms (model deformation)	Eyeball: 8638 E–1970 N Muscle: 2673 E-864 N Tetrahedral elements	CPU

Martínez et al. [36]	MD	Precomputation-based FEM (pre-comp FEM) using artificial neuro networks	The human breast	Hyperelastic (Mooney-Rivlin)	<0.2 s (model compression)	313,000 E-62,000 N Tetrahedral elements	2.6 GHz Intel (R) Xeon (R) CPU

Lorente et al. [8]	MD	Precomputation-based FEM (pre-comp FEM) using artificial neuro networks	The human liver	Nonlinear elasticity	2.89 s (model computing using machine learning) 51.63 s (model computing using FEM)	From 379,800 N to 420,690 N	3.4 GHz Intel Core i7, 8 GB RAM, OS X El Capitan

Tonutti et al. [7]	MD	Precomputation-based FEM (pre-comp FEM) using artificial neuro networks and support vector regression	The brain tumor	Nonlinear elasticity	<10 ms (model prediction using neural network)	6,442 N-1,087 E Tetrahedral elements	Core i7 2.9 GHz CPU

Luboz et al. [37]	MD	Precomputation-based FEM (pre-comp FEM) using the reduced order modelling method	The butt area	Nonlinear elasticity	<1 s (strain field computing)	27,649 E Hex-dominant elements	CPU

^∗^N: nodes; ^∗∗^NI: no information; ^∗∗∗^DOF: degree-of-freedom; ^∗∗∗∗^E: elements.

**Table 6:** Classification of developed modelling methods for soft tissue deformations in real time: meshfree-based techniques.

Reference	Approach	Modelling methods	Soft-tissue types	Tissue Behaviors	Computation time/speed	Geometry discretization	Hardware configurations
Nedel and Thalmann [38]	MD	Mass-spring system method (MSM)	The muscle	Linear elasticity	16 FPS (model deformation) 84 FPS (model deformation)	82 mass points 17 mass points	SGI Impact workstation, MIPS R10000 CPU

Monserrat et al. [4]	MD	Boundary element method (BEM)	The general cube mesh	Linear elasticity	15 Hz (model deformation)	<150 N Surface triangle elements	R-4400 CPU, 64 MB RAM

Goto and Lee [39]	MD	Statistical analysis method (SAM)-muscle	The human face	NI	1 minute (facial feature detection)	NI	Pentium II, 333 MHz CPU

Bonamico et al. [40]	MD	Mesh geometry VRML-like representation (VRML) & radial basis function (RBF)-muscle	The human face	Linear elasticity	475 ms (facial deformation) 1,430 ms (facial deformation)	1,253 V-2,444 F 4,152 V-8,126 F	Pentium II 450 MHz CPU, 128 MB RAM

Brown et al. [2]	MD	Mass-spring system method (MSM)	The blood vessel	Nonlinear viscoelasticity	24 FPS (system iteration) 6 FPS (system iteration)	216 N-1,440 E Surface triangle elements 8,000 N-66,120 E Surface triangle elements	Sun Ultra 60 Workstation 450 MHz CPU, 1 GB RAM

Sorkine et al. [41]	MD	Laplacian surface deformation (LSD)	The face model	Linear elasticity	0.07 s (model solving)	~10,000 V Surface triangle elements	2.0 GHz Pentium IV CPU

Chandrasiri et al. [42]	MD	Personal facial expression space method (PEES)-muscle	The human face	Linear elasticity	12 FPS (facial animation)	NI	1 GHz Athlon CPU

Mollemans et al. [43]	MD	Mass tensor method (MTM)	The cube The human face	Linear elasticity	From 24.57 s to 2.3 s	From 53,3380 N to 10,368 N Tetrahedral mesh	CPU

Chen et al. [44]	MD	Mass-spring system method (MSM) combined with quasistatic algorithm	The human brain	Linear elasticity	48 Hz–3,000 Hz (haptic feedback)	8,000 N Surface triangle elements	SGI Prism Server 4 GPU, 8 CPU, 32 GB RAM

López-Cano et al. [45]	MI	Mass-spring system method (MSM)	The human inguinal region	Linear elasticity	73 FPS (system iteration)	4,891 V Surface triangle elements	GPU NVIDIA 6,800, Pentium IV 3.0 GHz CPU, 1 GB RAM

Lim and De [46]	MD	Point collocation-based finite spheres (PCMFS)	The human liver	Nonlinear elasticity	1 ms (model deformation)	1,186 polygons Polygon elements	Pentium IV 2 GHz CPU, NVIDIA Quadro4 XGL

Murai et al. [5]	MD	Inverse dynamic computation (IDC)	The human muscles	Linear elasticity	16 ms (muscle tension estimation) 15 FPS (model rendering)	274 muscles	Intel Xeon 3.33 GHz CPU, 3.25 GB RAM, NVIDIA Quadro FX3700 GPU

Basafa and Farahmand [47]	MD	Mass-spring-damper method (MSD)	The cube model	Nonlinear viscoelasticity	5 ms (model deformation) 150 Hz (haptic feedback) 30 Hz (model rendering)	96 N-270 E Tetrahedral mesh 500 N Tetrahedral mesh	3.2 GHz Core Duo CPU, 1 GB RAM

Wang et al. [48]	MD	Laplacian surface deformation (LSD)	The human nose	Linear elasticity	NI	NI	Windows 2000 or Windows XP, 512 MB RAM or 250 MB

Ho et al. [6]	MD	Mass-spring system method (MSM)	The human eardrum	Linear elasticity	1 kHz (haptic feedback) 30 Hz (model rendering)	917 E Surface triangle elements	Intel Core2 Q6600 CPU, NVIDIA GeForce 9,600

Wan et al. [49]	MD	Radial basic function (RBF) & geodesic distance-muscle	The human face	Linear elasticity	0.0316 s (one system frame computing)	5,272 V-10,330 F Surface triangle elements	Intel Core 2 Duo E7200 2.53 GHz CPU, 2 GB RAM

Le et al. [50]	MD	Thin-shell deformation method (TSD)-muscle	The human face	Linear elasticity	73.8 FPM (facial animation) 164.3 FPM (facial animation)	40 markers 100 markers	Intel Xeon 2.4 GHz 16-Core CPU, NVIDIA Tesla C1060 240-Core GPU

Zhang et al. [51]	MD	Elastic-plus-muscle-distribution-based (E+MD)	The facial muscles	Linear elasticity	NI	NI	NI

Weng et al. [52]	MD	Facial motion regression algorithm (FMR)-muscle	The human face	NI	>200 FPS (graphic rendering on PC) 30 FPS (graphic rendering on mobile devices)	75 facial markers	Core i7 3.5 CPU Intel Atom 2.0 GHz CPU

Goulette and Chen [53]	MD	Hyperelastic mass link method for FEM (HEML-FEM)	The cube model	Viscohyperelasticity	4.02 ms (one model computation iteration) 21.24 ms (one model computation iteration)	4,430 E–1,128 N Tetrahedral mesh 21,436 E–5,591 N Tetrahedral mesh	Core 2 Duo 2.40 GHz CPU, 3.45 GB RAM

Zhang et al. [54]	MD	The time-saving volume-energy conserved ChainMail method (TSVE-Chainmail)	The cube model	Nonlinear elasticity	30 Hz (model rendering)	NI	Core i7-4700 3.4 GHz CPU

Woodward et al. [55]	MD	Radial basis function mapping approach (RBF)-muscle	The human face	Linear elasticity	2 minutes (system initializing) Up to 30 Hz (facial feature detection)	NI	NI

Zhou et al. [56]	MD	Marquardt radial basis meshless method (MRM)	The general cube model	Nonlinear elasticity	0.1509 s (model deformation)	121 nodes Tetrahedral mesh	Core i7-4790 3.60 GHz CPU, 8 GB RAM, Intel HD Graphics 4600 (64 MB)

**Table 7:** Classification of developed modelling methods for soft-tissue deformations in real time: combination-based techniques.

Reference	Approach	Modelling methods	Soft-tissue types	Tissue behaviors	Computation time/speed	Geometry discretization	Hardware configurations
Cotin et al. [57]	MD	Precomputation-based FEM (pre-comp FEM) & mass tensor method (MTM) & hybrid modelling method (HMM)	The blood vessel	Linear elasticity	40 Hz (model deformation) 500 Hz (haptic feedback)	760 vertices–4,000 edges 8,000 tetrahedral elements	233 MHz Dec Alpha Workstation

Yarnitzky et al. [58]	MD	Dynamics-based & FEM	The foot soft-tissue	Linear elasticity	<25 ms (model deformation)	100 nodes	1.6 GHz Dothan Pentium IV CPU, 1 GB RAM

Allard et al. [13]	MD	Multi-cooperative methods (multi-Corp)	NI	NI	NI	NI	NI

Zhu and Gu [59]	MD	Boundary element method (BEM) & mass-spring system (MSM) & particle surface interpolation (PSI)	The human liver	Linear elasticity with an extra mass-spring model	From 0.99 ms to 4.17 ms (model deformation)	From 200 to 1,200 nodes	2.26 GHz Pentium M CPU, GeForce 9650 M GPU, 2 GB RAM

## References

[1] Sun W., Lal P. (2002). Recent development on computer aided tissue engineering — a review. *Computer Methods and Programs in Biomedicine*.

[2] Brown J., Sorkin S., Latombe J.-C., Montgomery K., Stephanides M. (2002). Algorithmic tools for real-time microsurgery simulation. *Medical Image Analysis*.

[3] Peterlík I., Sedef M., Basdogan C., Matyska L. (2010). Real-time visio-haptic interaction with static soft tissue models having geometric and material nonlinearity. *Computers & Graphics*.

[4] Monserrat C., Meier U., Alcaniz M., Chinesta F., Juan M. C. (2001). A new approach for the real-time simulation of tissue deformations in surgery simulation. *Computer Methods and Programs in Biomedicine*.

[5] Murai A., Kurosaki K., Yamane K., Nakamura Y. (2010). Musculoskeletal-see-through mirror: computational modeling and algorithm for whole-body muscle activity visualization in real time. *Progress in Biophysics and Molecular Biology*.

[6] Ho A. K., Alsaffar H., Doyle P. C., Ladak H. M., Agrawal S. K. (2012). Virtual reality myringotomy simulation with real-time deformation: development and validity testing. *The Laryngoscope*.

[7] Tonutti M., Gras G., Yang G. Z. (2017). A machine learning approach for real-time modelling of tissue deformation in image-guided neurosurgery. *Artificial Intelligence in Medicine*.

[8] Lorente D., Martínez-Martínez F., Rupérez M. J. (2017). A framework for modelling the biomechanical behaviour of the human liver during breathing in real time using machine learning. *Expert Systems with Applications*.

[9] Delingette H. (1998). Toward realistic soft-tissue modeling in medical simulation. *Proceedings of the IEEE*.

[10] Nealen A., Müller M., Keiser R., Boxerman E., Carlson M. (2006). Physically based deformable models in computer graphics. *Computer Graphics Forum*.

[11] Moher D., Liberati A., Tetzlaff J., Altman D. G. (2010). Preferred reporting items for systematic reviews and meta-analyses: the PRISMA statement. *International Journal of Surgery*.

[12] Cotin S., Delingette H., Ayache N. (1999). Real-time elastic deformations of soft tissues for surgery simulation. *IEEE Transactions on Visualization and Computer Graphics*.

[13] Allard J., Cotin S., Faure F. (2007). SOFA - an open source framework for medical simulation. *Stud Health Technol Inform*.

[14] Berkley J., Turkiyyah G., Berg D., Ganter M., Weghorst S. (2004). Real-time finite element modeling for surgery simulation: an application to virtual suturing. *IEEE Transactions on Visualization and Computer Graphics*.

[15] Joldes G. R., Wittek A., Miller K. (2009). Suite of finite element algorithms for accurate computation of soft tissue deformation for surgical simulation. *Medical Image Analysis*.

[16] Miller K., Joldes G., Lance D., Wittek A. (2007). Total Lagrangian explicit dynamics finite element algorithm for computing soft tissue deformation. *Communications in Numerical Methods in Engineering*.

[17] Taylor Z. A., Cheng M., Ourselin S. (2008). High-speed nonlinear finite element analysis for surgical simulation using graphics processing units. *IEEE Transactions on Medical Imaging*.

[18] Audette M. A., Hayward V., Astley O., Doyon M., McCallister G. A., Chinzei K. (2004). A PC-based system architecture for real-time finite element-based tool-specific surgical simulation. *International Congress Series*.

[19] Sedef M., Samur E., Basdogan C. (2006). Real-time finite-element simulation of linear viscoelastic tissue behavior based on experimental data. *IEEE Computer Graphics and Applications*.

[20] Sela G., Subag J., Lindblad A., Albocher D., Schein S., Elber G. (2007). Real-time haptic incision simulation using FEM-based discontinuous free-form deformation. *Computer-Aided Design*.

[21] García M., Robles O. D., Pastor L., Rodríguez A. (2008). MSRS: a fast linear solver for the real-time simulation of deformable objects. *Computers & Graphics*.

[22] Joldes G. R., Wittek A., Miller K. (2008). An efficient hourglass control implementation for the uniform strain hexahedron using the total Lagrangian formulation. *Communications in Numerical Methods in Engineering*.

[23] Joldes G. R., Wittek A., Miller K. (2010). Real-time nonlinear finite element computations on GPU – application to neurosurgical simulation. *Computer Methods in Applied Mechanics and Engineering*.

[24] Wittek A., Joldes G., Couton M., Warfield S. K., Miller K. (2010). Patient-specific non-linear finite element modelling for predicting soft organ deformation in real-time; application to non-rigid neuroimage registration. *Progress in Biophysics and Molecular Biology*.

[25] Lapeer R. J., Gasson P. D., Karri V. (2010). Simulating plastic surgery: from human skin tensile tests, through hyperelastic finite element models to real-time haptics. *Progress in Biophysics and Molecular Biology*.

[26] Marchesseau S., Heimann T., Chatelin S., Willinger R., Delingette H. (2010). Fast porous visco-hyperelastic soft tissue model for surgery simulation: application to liver surgery. *Progress in Biophysics and Molecular Biology*.

[27] Courtecuisse H., Jung H., Allard J., Duriez C., Lee D. Y., Cotin S. (2010). GPU-based real-time soft tissue deformation with cutting and haptic feedback. *Progress in Biophysics and Molecular Biology*.

[28] Turkiyyah G. M., Karam W. B., Ajami Z., Nasri A. (2011). Mesh cutting during real-time physical simulation. *Computer-Aided Design*.

[29] Niroomandi S., Alfaro I., Cueto E., Chinesta F. (2012). Accounting for large deformations in real-time simulations of soft tissues based on reduced-order models. *Computer Methods and Programs in Biomedicine*.

[30] Wu T., Hung A. P. L., Hunter P., Mithraratne K. (2013). Modelling facial expressions: a framework for simulating nonlinear soft tissue deformations using embedded 3D muscles. *Finite Elements in Analysis and Design*.

[31] Morooka K., Nakasuka Y., Kurazume R., Chen X., Hasegawa T., Hashizume M. Navigation system with real-time finite element analysis for minimally invasive surgery.

[32] Mafi R., Sirouspour S. (2014). GPU-based acceleration of computations in nonlinear finite element deformation analysis. *International Journal for Numerical Methods in Biomedical Engineering*.

[33] Courtecuisse H., Allard J., Kerfriden P., Bordas S. P. A., Cotin S., Duriez C. (2014). Real-time simulation of contact and cutting of heterogeneous soft-tissues. *Medical Image Analysis*.

[34] Strbac V., Vander Sloten J., Famaey N. (2015). Analyzing the potential of GPGPUs for real-time explicit finite element analysis of soft tissue deformation using CUDA. *Finite Elements in Analysis and Design*.

[35] Karami A., Eghtesad M., Haghpanah S. A. (2017). Prediction of muscle activation for an eye movement with finite element modeling. *Computers in Biology and Medicine*.

[36] Martínez-Martínez F., Rupérez-Moreno M. J., Martínez-Sober M. (2017). A finite element-based machine learning approach for modeling the mechanical behavior of the breast tissues under compression in real-time. *Computers in Biology and Medicine*.

[37] Luboz V., Bailet M., Boichon Grivot C. (2018). Personalized modeling for real-time pressure ulcer prevention in sitting posture. *Journal of Tissue Viability*.

[38] Nedel L. P., Thalmann D. Real time muscle deformations using mass-spring systems.

[39] Goto T., Lee W. S., Magnenat-thalmann N. (2002). Facial feature extraction for quick 3D face modeling. *Signal Processing: Image Communication*.

[40] Bonamico C., Costa M., Lavagetto F., Pockaj R. (2002). Real-time MPEG-4 facial animation with 3D scalable meshes. *Signal Processing: Image Communication*.

[41] Sorkine O., Cohen-Or D., Lipman Y., Alexa M., Rössl C., Seidel H.-P. Laplacian surface editing.

[42] Chandrasiri N. P., Naemura T., Ishizuka M., Harashima H., Barakonyi I. (2004). Internet communication using real-time facial expression analysis and synthesis. *IEEE Multimedia*.

[43] Mollemans W., Schutyser F., Nadjmi N., Suetens P. (2005). Very fast soft tissue predictions with mass tensor model for maxillofacial surgery planning systems. *International Congress Series*.

[44] Chen P., Barner K. E., Steiner K. V. A displacement driven real-time deformable model for haptic surgery simulation.

[45] López-Cano M., Rodríguez-Navarro J., Rodríguez-Baeza A., Armengol-Carrasco M., Susín A. (2007). A real-time dynamic 3D model of the human inguinal region for surgical education. *Computers in Biology and Medicine*.

[46] Lim Y. J., De S. (2007). Real time simulation of nonlinear tissue response in virtual surgery using the point collocation-based method of finite spheres. *Computer Methods in Applied Mechanics and Engineering*.

[47] Basafa E., Farahmand F. (2011). Real-time simulation of the nonlinear visco-elastic deformations of soft tissues. *International Journal of Computer Assisted Radiology and Surgery*.

[48] Wang J., Liao S., Zhu X. (2011). Real time 3D simulation for nose surgery and automatic individual prosthesis design. *Computer Methods and Programs in Biomedicine*.

[49] Wan X., Liu S., Chen J. X., Jin X. (2012). Geodesic distance-based realistic facial animation using RBF interpolation. *Computing in Science & Engineering*.

[50] Le B. H., Zhu M., Deng Z. (2013). Marker optimization for facial motion acquisition and deformation. *IEEE Transactions on Visualization and Computer Graphics*.

[51] Zhang Y., Lin W., Zhou B., Chen Z., Sheng B., Wu J. (2014). Facial expression cloning with elastic and muscle models. *Journal of Visual Communication and Image Representation*.

[52] Weng Y., Cao C., Hou Q., Zhou K. (2014). Real-time facial animation on mobile devices. *Graphical Models*.

[53] Goulette F., Chen Z.-W. (2015). Fast computation of soft tissue deformations in real-time simulation with hyper-elastic mass links. *Computer Methods in Applied Mechanics and Engineering*.

[54] Zhang J., Zhong Y., Smith J., Gu C. (2016). A new ChainMail approach for real-time soft tissue simulation. *Bioengineered*.

[55] Woodward A., Chan Y. H., Gong R. (2017). A low cost framework for real-time marker based 3-D human expression modeling. *Journal of Applied Research and Technology*.

[56] Zhou J., Luo Z., Li C., Deng M. (2018). Real-time deformation of human soft tissues: A radial basis meshless 3D model based on Marquardt's algorithm. *Computer Methods and Programs in Biomedicine*.

[57] Cotin S., Delingette H., Ayache N. (2000). A hybrid elastic model for real-time cutting, deformations, and force feedback for surgery training and simulation. *The Visual Computer*.

[58] Yarnitzky G., Yizhar Z., Gefen A. (2006). Real-time subject-specific monitoring of internal deformations and stresses in the soft tissues of the foot: a new approach in gait analysis. *Journal of Biomechanics*.

[59] Zhu B., Gu L. (2012). A hybrid deformable model for real-time surgical simulation. *Computerized Medical Imaging and Graphics*.

[60] Pandzic I. S., Forchheimer R. (2003). *MPEG-4 Facial Animation: The Standard, Implementation and Applications*.

[61] Doblaré M., Cueto E., Calvo B., Martínez M. A., Garcia J. M., Cegoñino J. (2005). On the employ of meshless methods in biomechanics. *Computer Methods in Applied Mechanics and Engineering*.

[62] Zhang G. Y., Wittek A., Joldes G. R., Jin X., Miller K. (2014). A three-dimensional nonlinear meshfree algorithm for simulating mechanical responses of soft tissue. *Engineering Analysis with Boundary Elements*.

[63] Doweidar M. H., Calvo B., Alfaro I., Groenenboom P., Doblaré M. (2010). A comparison of implicit and explicit natural element methods in large strains problems: application to soft biological tissues modeling. *Computer Methods in Applied Mechanics and Engineering*.

[64] Roohbakhshan F., Sauer R. A. (2017). Efficient isogeometric thin shell formulations for soft biological materials. *Biomechanics and Modeling in Mechanobiology*.

[65] Kamensky D., Xu F., Lee C. H., Yan J., Bazilevs Y., Hsu M. C. (2018). A contact formulation based on a volumetric potential: application to isogeometric simulations of atrioventricular valves. *Computer Methods in Applied Mechanics and Engineering*.

[66] Sørensen T. S., Mosegaard J., Harders M., Székely G. (2006). An introduction to GPU accelerated surgical simulation. *Biomedical Simulation*.

[67] Dao T. T., Fan A.-X., Dakpé S., Pouletaut P., Rachik M., Tho M. C. H. B. (2018). Image-based skeletal muscle coordination: case study on a subject specific facial mimic simulation. *Journal of Mechanics in Medicine and Biology*.

[68] Dao T. T., Tho M.-C. H. B. (2018). A systematic review of continuum modeling of skeletal muscles: current trends, limitations, and recommendations. *Applied Bionics and Biomechanics*.

[69] Wan B., Shahmoradi M., Zhang Z. (2019). Modelling of stress distribution and fracture in dental occlusal fissures. *Scientific Reports*.

[70] Zhang Y., Liu Y., She Y., Liang Y., Xu F., Fang C. (2019). The effect of endodontic access cavities on fracture resistance of first maxillary molar using the extended finite element method. *Journal of Endodontia*.

[71] Niroomandi S., Alfaro I., González D., Cueto E., Chinesta F. (2012). Real-time simulation of surgery by reduced-order modeling and X-FEM techniques. *International Journal for Numerical Methods in Biomedical Engineering*.

[72] Avazmohammadi R., Li D. S., Leahy T. (2018). An integrated inverse model-experimental approach to determine soft tissue three-dimensional constitutive parameters: application to post-infarcted myocardium. *Biomechanics and Modeling in Mechanobiology*.

[73] Avazmohammadi R., Soares J. S., Li D. S., Raut S. S., Gorman R. C., Sacks M. S. (2019). A contemporary look at biomechanical models of myocardium. *Annual Review of Biomedical Engineering*.

